# Assignment of low-molecular-weight selenometabolites in the root section of white cabbage

**DOI:** 10.1007/s00425-025-04651-y

**Published:** 2025-03-01

**Authors:** Áron Soós, Béla Kovács, Tünde Takács, Márk Rékási, Péter Dobosy, Csaba Szőke, Mihály Dernovics, Péter Ragályi

**Affiliations:** 1 Institute of Food Science, Faculty of Agricultural and Food Sciences and Environmental Management, University of Debrecen, Böszörményi út 138, Debrecen, 4032 Hungary; 2 Institute for Soil Sciences, HUN-REN Centre for Agricultural Research, Fehérvári út 132-144, Budapest, 1116 Hungary; 3 Institute of Aquatic Ecology, HUN-REN Centre for Ecological Research, Karolina út 29, Budapest, 1113 Hungary; 4 Department of Maize Breeding, Agricultural Institute, HUN-REN Centre for Agricultural Research, Brunszvik u. 2, Martonvásár, 2462 Hungary; 5 Department of Plant Physiology and Metabolomics, Agricultural Institute, HUN-REN Centre for Agricultural Research, Brunszvik u. 2, Martonvásár, 2462 Hungary

**Keywords:** Brassicaceae, Cabbage root, ESI–MS, Hyphenated systems, Selenate fertilization, Selenium recovery

## Abstract

**Main conclusion:**

Quantitative and qualitative selenium speciation analyses of the root of white cabbage reveal the presence of elemental Se, selenate, selenomethionine and deaminated derivatives of selenohomolanthionine.

**Abstract:**

White cabbage (*Brassica oleracea* convar. *capitata* var. *alba*) is one of the most consumed vegetable brassicas of the *Brassica oleracea* species whose production is compatible with the recent strip-till and no-till type farming policies. White cabbage has been in the focus of selenium research for decades as a possible source of food-derived selenium supplementation; however, the root section of the plant has hardly been targeted, being a by-product that is left in or plowed into the soil to serve as an organic fertilizer. The root of selenium-enriched white cabbage, planted on three different soil types (sand, silty sand, and silt), was analyzed for selenium speciation with the complementary use of liquid chromatography inductively coupled plasma mass spectrometry (LC–ICP-MS) and electrospray ionization high-resolution mass spectrometry (LC–ESI–HR-MS) methods after orthogonal (anion/cation exchange) chromatographic purification. Elemental selenium (Se^0^) was the major selenospecies in all cases, accounting for 28–43% of total selenium content. Water and proteolytic extractions could recover a median of 28% of total selenium through the quantification of selenate and selenomethionine, leaving a series of selenocompounds unassigned. Among these latter species, accounting for up to an additional 6% of total selenium, eight low-molecular-weight selenocompounds were detected; five out of the eight compounds could be tentatively identified as deaminated derivatives of selenohomolanthionine.

**Supplementary Information:**

The online version contains supplementary material available at 10.1007/s00425-025-04651-y.

## Introduction

Analytical tasks encountered in the case of phytoremediation, biofortification, and accumulation or enrichment studies on selenium evidently include the determination of elemental concentrations. When applicable, e.g., in pot size experiments or in reference material production, full recovery, or, in other terms, full coverage of the given element, is targeted and can be obtained to map elemental distribution, enrichment/accumulation/volatilization factors, and to determine quantitative mass balance (Pilon-Smits et al. 1999; Li et al. 2021a; Pereira et al. 2022). In turn, full recovery on the level of elemental species is hardly achieved, and it highly depends on the actual element and sample matrix. When the number of elemental species is limited and their extraction is straightforward, high recovery (> 90%) can be targeted, which is often reported for arsenic speciation (Mihucz et al. 2005; Gajdosechova et al. 2020).

In the case of selenium speciation, a huge set of commercially unavailable, unknown, or even formerly undetected species with different biochemical properties can be present in a sample, which makes full species coverage, i.e., quantification, practically impossible. Even the most characterized selenized sample, selenium-enriched yeast SELM-1 certified reference material, where almost a hundred species have just been listed (LeBlanc and Mester 2021), could not be quantified on each selenium species. To achieve this without authentic standards, liquid chromatography–post-column isotope dilution analysis–inductively coupled plasma mass spectrometry (LC–IDA–ICP-MS) can be properly addressed, but the high number of species hampers this due to chromatographic resolution and species intensity issues (García-Sevillano et al. 2013). A possible intermediate solution is to target the most concentrated selenospecies only that are highly abundant enough to stay at a quantifiable concentration after dilution, which decreases the low abundant species under the actual limit of quantification. In such cases, the use of IDA or quantifying with the help of a close-eluting selenocompound can give fit-for-purpose results (Dernovics et al. 2007; Ouerdane et al. 2020).

However, in the long run, the analytical focus cannot be limited to the most studied selenospecies, namely, selenomethionine (SeMet), selenocysteine, methylselenocysteine (MetSeCys), selenate, and selenite. The reason behind the fact that these are the most reported species is that these are commercially available, which often leads to forced and incorrect identification issues, especially in the case of selenocysteine (Dernovics 2024). The diversity of selenospecies is not only wide but extends continuously with novel species that turn out to be major contributors to the selenium mass balance, even in phylogenetically highly unrelated entities. For example, selenohomolanthionine was first reported to be only the third most concentrated water-soluble selenocompound in Japanese pungent radish (*Raphanus sativus* L. cv. 'Yukibijin') after selenate and MetSeCys (Ogra et al. 2007), and then, it proved to be the dominating main species in selenium-enriched Torula yeast (*Candida utilis*) (Bierła et al. 2017). Similarly, γ-glutamyl-selenomethionine was unambiguously identified first in garlic (*Allium sativum*, Larsen et al. 2006) as a relatively low-level selenocompound and detected later as the major selenospecies in the probiotic *Bifidobacterium longum* DD98 (Zhu et al. 2022). All these validated identifications could be carried out due to the involvement of electrospray ionization high-resolution mass spectrometry (ESI–HR-MS). This technology is far less exposed to chromatographic resolution limitations compared to ICP-MS but suffers, among other things, from highly matrix-dependent sensitivity, vendor- and ion source-related ionization/fragmentation behavior, and restricted use of solvents/eluents. Still, apart from volatile selenometabolites, LC-ESI–HR-MS is the key instrumentation to explore the complexity of selenium speciation with the possibility to offer accurate elemental composition data and at least tentative structural identification, which turns into crucial when carrying out retrospective analysis to re-evaluate former assignments. For example, the non-proteinaceous selenoamino acid selenolanthionine was first indirectly assigned in selenized yeast with the help of ion-pairing LC–ICP-MS hyphenation (Kotrebai et al. 2000), but it has never been detected afterward in any yeast samples but as the major selenium form in the selenium accumulator plant *Cardamine violifolia* (Both et al. 2018). This latter case, in-house synthesis of selenolanthionine was a key demand to validate the identification and to quantify this compound—nonetheless, analytical laboratories cannot be required to synthesize each commercially unavailable compound detected in their samples.

A practical “trade-off” approach can be the combination of targeting the main selenospecies with LC–ICP-MS, providing a considerable quantitative recovery of selenometabolites, and assigning the directly non-LC–ICP-MS amenable (that is, unknown or less concentrated) selenometabolites through LC-ESI–HR-MS analyses (Egressy-Molnár et al. 2016). Such a method helps gain a deeper insight into selenium metabolism and unearth intermediate-level selenometabolites whose concentration might reach that of the main selenocompounds in a completely different sample or in the same sample type under different cultivation circumstances. Linked to the latter case, it is to highlight the effect of soil microbiome: indeed, there has been a high concern about the possible contribution of soil microorganisms to the uptake, transformation, and even recirculation of selenium. Accordingly, selenocompounds detected in plants, or more specifically, in the root system, can be a function of both surrounding (bulk soil and rhizosphere) and endophytic microbiomes (Cochran et al. 2018; Li et al. 2021b).

Our study was aiming at the root section of white cabbage (*Brassica oleracea* convar. *capitata* var. *alba*), a plant of the Brassicaceae family whose production is compatible with the recent trends of strip-till and no-till farming methods (Übelhör et al. 2014; Thomazini et al. 2015). White cabbage is the most produced species out of the general vegetable cabbage set, exceeding 82 million tons/year, harvested from more than 2.8 million hectares (Shokirov et al. 2021). In the EU, it was the third most exported vegetable in 2022 on a price basis (0.36 € billion) (Eurostat 2024). This plant has been long known for its selenium accumulation potential due to its complex sulfur chemistry (Zayed and Terry 1994; Peñas et al. 2012) and it has been a common model plant of selenium biochemistry since the 70's (Hamilton 1975). Still, its selenium speciation coverage is very limited to only a few studies. In the publication of Slekovec and Goessler (2005), cabbage accumulated the highest amount of Se (up to 12 mg kg^−1^ DW) among the plant species involved, but the authors reported that only 20% of total Se could be extracted when addressing methanol/water solution for the extraction, and 90% of extracted Se was in the form of selenate.

From the physiological point of view, a special attention has been made to the root system of cabbage: while both inorganic and organic selenometabolites have been detected (Mechora et al. 2012; Ragályi et al. 2024) there, referring to a considerable biotransformation capacity, the transfer of selenium from the root toward the stem and leaves is the lowest among other vegetable Brassica species (Wang et al. 2023). This fact refers to either an at least partly selective or tissue-specific species transport, or the influence of local microbiomes.

Our main goal was to provide the highest possible selenium coverage of the root sample. To achieve this, the combined use of orthogonal (two-dimensional) liquid chromatography (LC)-assisted purification prior to ESI–HR-MS-based species assignment and the quantification of the major selenometabolites through LC–ICP-MS were applied. Besides organic selenocompounds, the extraction and quantification of elemental Se (Se^0^) were also targeted, to include this relatively rarely covered selenium species in the selenium mass balance.

## Materials and methods

### Cultivation parameters

Cabbage (*Brassica oleracea* convar. *capitata* var. *alba* cv. Zora) was cultivated in a greenhouse on the research station of the Institute for Soil Sciences, Centre for Agricultural Research, HUN-REN (Őrbottyán, Hungary) in 2019, as presented by Ragályi et al. (2024), and only a short description is provided here. Before the pot experiment, cabbage seeds were germinated and planted in propagation trays (1 seed per cell) filled with “VEGASCA Bio” (Florasca Hungary Ltd., Osli, Hungary) growing medium (peat and gray cattle manure compost with the following characteristics: organic matter > 50%; N > 0.3%; P_2_O_5_ > 0.1%; K_2_O > 0.1%; pH: 6.8). After a 6-day acclimatization period in the greenhouse, the soil-free seedlings were transplanted into the experimental pots at 1 seedling per pot rate. The plants were grown in 10-L pots filled with the homogenized top layer (0–20 cm) of three soils with different textures: sand (Mollic Umbrisol, Arenic, from Őrbottyán, 47 °40 ′N, 19 °14 ′E), silty sand (Luvic Calcic Phaeozem from Gödöllő, 47°58 ′N, 19°38 ′E), and silt (Calcic Chernozem from Hatvan, 47 °67 ′N, 19 °64 ′E). The basic characteristics of the soils are shown in Supplementary Table S1. The climatic conditions of the greenhouse were continuously monitored during the growing period. The mean day and night temperatures were 25.5 ± 4.0 and 18.2 ± 3.4 °C, respectively. The air humidity was 72.2 ± 23.0%. Pesticide “Decis” (Bayer AG, Leverkusen, Germany) was applied whenever necessary. The nutrient requirement of cabbage was covered with Hoagland solution (undiluted; 200 mL per pot) during the vegetation period. Irrigation was carried out with an automated system using individual drip stakes placed in each pot. Soil moisture was maintained at a level of 22 ± 6% v/v.

After planting, tap water was applied without Se treatment uniformly throughout the whole experiment for the first three weeks. The Se content of the tap water was 0.287 ± 0.044 μg Se L^−1^. Irrigation of the treated pots with a 561 ± 49 µg Se L^−1^ (actual concentration) solution in the form of Na_2_SeO_4_ diluted in tap water started 3 weeks after planting. The experiment was set up with three replicates. The growing period of the cabbage, from transplanting to harvest, lasted from July 17 to September 25. During this time, each plant received 19,065 mL of irrigation water treated with Se, that is, a total of 10.70 mg of Se per pot.

### Chemical analysis

After harvest, the plants were washed with deionized water, the roots and the aboveground parts were separated, and the fresh weight was measured. The roots were dried at 40 °C for two days in a laboratory oven, followed by the determination of dry mass. Dried samples were homogenized in a household blending machine equipped with a plastic housing and a stainless-steel blade. The dried, homogenized samples were kept in centrifuge tubes in a dark room at room temperature until analysis.

For the analysis of soil samples, organic matter (OM) content was determined according to the FAO 2020 method (FAO 2020); cation exchange capacity (CEC) according to ISO 13536:1995; ammonium-lactate-soluble potassium (AL-K) and phosphorus (AL-P) according to Egnér et al. (1960); total potassium and phosphorus according to ISO 12914:2012; ammonium acetate + EDTA-soluble (LE) selenium according to Lakanen and Erviö (1971); water-soluble Se according to the Hungarian Standard Method MSZ 21470–50:2006; CaCO_3_ content according to ISO 10693:1997); total N content according to ISO 11261:1995; nitrate content according to Thamm (1990).

For total Se determination, the dried, homogenized samples were mineralized in a microwave-assisted acid digestion system (TopWave, Analytik Jena, Jena, Germany). Dried samples (400–500 mg) were digested in a mixture of 7 mL 67% HNO_3_ and 3 mL 30% H_2_O_2_. After digestion, the internal standards were added to the solutions, and the volume was made up to 15 mL with deionized water. The concentrations of Se and other elements in the soil and plant samples were measured with an inductively coupled plasma mass spectrometer (ICP-MS, PlasmaQuant Elite, Analytik Jena) with a determination precision of 1–3% relative standard deviation (RSD) for Se.

### Sample preparation for selenium speciation and for fraction collection

For the sample preparation and quantification of SeMet, MetSeCys and Se VI (Merck-Sigma Group, Darmstadt, Germany), the methods published in the study of Shao et al. (2014) and in the Annex of the Commission Implementing Regulation (EU 2020/2117) were taken and adapted. In short, dried and homogenized samples were further milled with an MM 200 mixer mill (Retsch, Haan, Germany). About 0.25 g of cabbage root samples were weighed into 15-mL centrifuge tubes with 10 mL deionized water (Millipore, Molsheim, France) and were sonicated for 10 min. Afterwards, the samples were centrifuged at 8000 *g* for 10 min, and then, the supernatants were filtered through a 0.22 µm pore-sized hydrophilic poly(tetrafluoroethylene) (PTFE) syringe filter. To prevent the oxidation, 0.01% dithiothreitol (Merck-Sigma Group) was added and the samples were subjected to the strong anion exchange (SAX)-ICP-MS for quantitative analysis.

The solid residues were either enzymatically digested with Pronase E (from *Streptomyces griseus*, 4,000,000 PU g^−1^, Merck-Sigma Group) in a water bath at 37 °C overnight or were subjected to Na_2_SO_3_ extraction (see later). About 40 mg enzyme per sample was dissolved in 9.0 mL 0.1 M TRIS buffer (pH = 9, adjusted with 1.0 M HCl solution; Merck-Sigma Group) and was mixed with the plant residues. An additional 40 mg of pronase enzyme per sample was dissolved in 1.0 mL TRIS buffer and was added to the samples the following day, and the samples were shaken for an additional period of six hours at 37 °C. Samples were then centrifuged at 8000 *g* for 10 min. Supernatants were removed and filtered through a 0.22 µm pore-sized hydrophilic PTFE syringe filter. To prevent the oxidation of SeMet, 0.01% dithiothreitol was added and the samples were subjected to the HPLC(SAX)–ICP-MS for quantitative analysis.

Elemental selenium (Se^0^) was extracted and quantified according to Both et al. (2018). Briefly, water-insoluble residues obtained after water extraction were extracted with 5.0 mL of 1.0 M sodium sulfite (Merck-Sigma Group) solution with shaking in a water bath at 37 °C overnight and then centrifuged at 8000 *g* for 10 min. After removing the supernatants, 4 mL of deionized water was pipetted to the residues. The samples were vortexed for one minute and were again centrifuged at 8000 *g* for 10 min. The supernatants were pooled and filled up to 10 mL with deionized water. Five ml of the Na_2_SO_3_ extracted solutions was pipetted into PTFE vessels, and then mixed with 7 mL nitric acid (65% w/w) and 1 mL hydrogen peroxide (30% w/w) from Scharlab S.L. (Sentmenat, Spain). The digestion was performed in a microwave-assisted digestion system (Milestone Start D, Sorisole, Italy) at 180 °C. ICP-MS (X-Series II, Thermo Scientific, Bremen, Germany) was used for the quantification with the standard addition method, taking Rh as the internal standard and correcting with the inherent selenium content of the sodium sulfite reagent. Instrument parameters were the same as those given for Se speciation with HPLC(SAX)–ICP-MS.

### Se speciation by HPLC(SAX)–ICP-MS

Species in the water extracts and in the enzymatic extracts were separated by SAX chromatography and detected using ICP-MS (Ragályi et al. 2024). A Waters Alliance 2695 HPLC instrument (Waters Co., Milford, MA, USA) was coupled to an X-Series II ICP-MS (Thermo Scientific). The injection volume was 10 µL for the enzymatic extracts, while for the water extracts, 20 µL was set to fit the analyte concentration and possible matrix interferences. A Hamilton PRP-X100 (250 × 4.1 mm, 10 µm) SAX column (Hamilton Company, Reno, NV, USA) was used at a temperature of 25 °C. Gradient elution was done with ammonium acetate (pH = 6.5, eluent A: 10 mM, eluent B: 250 mM) at a 2.0 mL min^−1^ flow rate. Eluents were prepared from ammonium acetate (ACS, Reag. Ph. Eur., ≥ 98%, Merck-Sigma Group), and the pH was adjusted with acetic acid (glacial; ACS, Reag. Ph. Eur., VWR, Radnor, PA, USA). The program was as follows: 0–1 min: 0% B, 1–20 min: linear gradient up to 100% B, 20–29 min: 100% B, 29–31 min: linear gradient down to 0% B, 31–37 min: 0% B. Quantification of MetSeCys was targeted in separate injections with a modified LC gradient (0–1 min: 0% B, 1–2 min: linear gradient up to 5% B, 2–3 min: linear gradient up to 100% B, 3–10 min: 100% B, 10–11 min: linear gradient down to 0% B, 11–18 min: 0% B).

The main ICP-MS parameters were as follows: forward power 1400 W, plasma gas flow rate 14.0 L min^−1^, nebulizer gas flow rate 0.86 L min^−1^, and auxiliary gas flow rate 0.88 L min^−1^. H_2_–He (in 7:93%) collision cell gas was applied with a 6.0 mL min^−1^ flow rate. The detected ions were ^78^Se and ^80^Se, but ^78^Se was used for quantification. Standard addition was applied for the quantification of MetSeCys, SeMet, and Se VI. The concentration of selenocompounds eluted as unassigned peaks was estimated with the help of the calibration curve recorded for SeMet. Concentrations are expressed in dry mass, related to selenium.

### Orthogonal liquid chromatography-based fraction collection of selenometabolites for UPLC–Unispray-QTOF-MS analysis

The water extracts obtained from the root samples of all three types of soils were pooled, lyophilized, and redissolved in the starting SAX eluent (see later) to obtain a concentrated extract for the fraction collection process. The separation was done with the Waters Alliance 2695 HPLC instrument using strong anion exchange (SAX) and strong cation exchange (SCX) chromatographic setups. The analytical columns were thermostated at 25 °C. The injection volume was 100 µL. The LC effluents were introduced into the X-Series II ICP-MS to monitor the elution of Se-containing compounds on the ^78^Se isotope and to determine the retention time frames. For fraction collection purposes, the effluent was disconnected from the ICP-MS, and the fractions were collected manually. Retention time frames were checked on a daily basis during the whole fraction collection campaign.

At the first step of the purification, SAX chromatography was applied on the PRP-X100 column (250 mm × 4.1 mm, 10 μm). Eluent A was 10 mM ammonium acetate (pH = 5.5), while eluent B was 300 mM ammonium acetate (pH = 5.5). Gradient elution was applied as follows: 0 min: 0% B, 0–8.5 min: linear gradient up to 85% B, 8.5–8.6 min: linear gradient down to 0% B, 8.6–11.5 min: 0% B. Flow rate was 2.0 mL min^−1^.

After fraction collection, the same fractions from the three chosen samples were pooled (CR SAX1-7) and lyophilized, and then, the residues were dissolved in 1.0 or 2.0 mL of 1 mM pyridine formate (pH = 2.8), depending on the concentration of Se species. Pyridine was obtained from Spectranal (Honeywell Riedel-De Haёn AG, Seelze, Germany), and the pH was adjusted with formic acid (98–100%, ACS, Reag. Ph. Eur.; Scharlab S.L.). The solutions were filtered (0.22 µm hydrophilic poly(tetrafluoroethylene) (PTFE), and then, the purification was continued with SCX chromatography on a PRP-X200 column (250 mm × 4.1 mm, 10 μm; Hamilton), according to Domokos-Szabolcsy et al. (2024). Eluent A was 1 mM pyridine formate (pH = 2.8), while eluent B was 40 mM pyridine formate (pH = 2.8; the concentration refers to pyridine). The gradient program was as follows: 0–1 min: 0% B at 1.7 mL min^−1^; 1–15 min: linear gradient up to 30% B at 1.7 mL min^−1^; 15–16 min: linear gradient up to 100% B at 1.9 mL min^−1^; 16–21 min: 100% B at 1.9 mL min^−1^; 21–21.5 min: linear gradient down to 0% B at 1.7 mL min^−1^; 21.5–26 min: 0% B at 1.7 mL min^−1^.

Fractions were lyophilized (Scanvac Coolsafe 55–4; Labogene, Lillerød, Denmark) and then dissolved in 0.25 mL of deionized water containing 0.1% V/V formic acid for the UPLC–Unispray-QTOF-MS analyses.

A Vion ion mobility quadrupole time-of-flight mass spectrometer (Waters) equipped with a UniSpray (Waters) ion spray source was applied. Chromatographic elution was provided by an Acquity UPLC I-Class system (Waters) using a BEH-C18 reversed phase (RP) UPLC column (100 mm × 2.1 mm, 1.7 μm; Waters). Chromatographic elution with eluent A (deionized water with 0.1% v/v formic acid /LC–MS grade; VWR/) and eluent B (acetonitrile /LC–MS grade, VWR/ with 0.1% v/v formic acid) was carried out at 0.4 mL min^−1^ with two different gradients (#1 and #2) to decrease the co-elution of species. The related instrumental and gradient parameters are described in the Supplementary material (Tables S2, S3). Selenium-containing species were screened in the full-scan spectra according to the method developed by Ouerdane et al. (2020) and they were subjected to MS/MS analysis with an individually optimized fragmentation energy setting.

## Results and discussion

### Quantification of total Se, elemental selenium, methylselenocysteine, selenomethionine, and selenate

The root section of cabbage plants has hardly been investigated from a selenium speciation point of view, and accordingly, to the best of our knowledge, there has been only one study dealing with non-foliar type selenate fertilization. Mechora et al. (2012) conducted an experiment on red cabbage (*Brassica oleracea* var. *capitate* L. f. *rubra*) where soil fertilization was applied twice with 0.5 mg L^−1^ selenate solution to the plants. The authors reported that 0.33 mg Se kg^−1^ DW out of 1.19 mg Se kg^−1^ DW, that is, 28% of total Se in the roots, could be extracted with proteolytic digestion, detecting selenomethionine as the solely present selenocompound, which indirectly indicated the complete biotransformation of the selenate uptaken by the plants at this level of fertilization. Indeed, selenate could have been co-extracted with selenomethionine during the sample preparation process, but the authors found this inorganic species only in traces, under the limit of quantification. Selenomethionine finally contributed to 15% of the total Se content of the roots, leaving 85% as non-extracted and/or unidentified.

In our case, the higher level selenate fertilization contributed to a significantly higher total selenium concentration in the roots (Table 1; 51.0–62.1 mg kg^−1^ DW), which still did not provoke any visible sign of stress in the plants. There was a significant difference between the total Se content of roots sampled from sand and silt, favoring the former type of soil in terms of selenium uptake.

**Table 1:** Quantification of total selenium, selenomethionine (SeMet), selenate (Se VI), methylselenocysteine (MetSeCys), elemental Se, total nitrogen, total nitrate nitrogen, and total protein nitrogen (calculated as the difference between total nitrogen and total nitrate nitrogen contents) in cabbage root samples and extracts

Sample label	Total selenium content; mg kg^−1^		Selenospecies extracted with water; expressed as mg Se kg^−1^			Selenospecies extracted with enzymatic digestion; expressed as mg Se kg^−1^			Summed recovery of selenium through water extraction and enzymatic digestion; expressed as mg Se kg^−1^ and percentage	Elemental Se (Se^0^) content, expressed as mg Se kg^−1^ and percentage	Overall Se speciation recovery	Total N content, g kg^−1^	Nitrate N content, g kg^−1^	Protein N content, g kg^−1^
			SeMet	Se VI	MetSeCys	SeMet	Se VI	MetSeCys						
Sand 1	57.3	59.4 ± 2.4^a^	< LOQ	11.9	< LOQ	14.7	2.1	< LOQ	28.7 (50%)	22.0 (37%)	87%	20.8	0.6	20.2
Sand 2	62.1		< LOQ	3.4	< LOQ	12.0	< LOQ	< LOQ	15.4 (25%)	18.7 (30%)	55%	8.66	0.22	8.44
Sand 3	58.7		< LOQ	3.0	< LOQ	10.4	< LOQ	< LOQ	13.4 (23%)	21.7 (37%)	60%	12.5	0.1	12.4
Silt 1	52.8	52.6 ± 1.5^b^	< LOQ	2.6	< LOQ	10.3	< LOQ	< LOQ	12.9 (24%)	17.5 (33%)	57%	14.7	0.1	14.6
Silt 2	53.9		< LOQ	4.0	< LOQ	13.6	< LOQ	< LOQ	17.6 (33%)	20.1 (37%)	69%	14.6	0.2	14.4
Silt 3	51.0		< LOQ	3.3	< LOQ	10.8	< LOQ	< LOQ	14.1 (28%)	20.0 (28%)	56%	12.8	0.2	12.6
Silty sand 1	56.5	54.6 ± 1.9^ab^	< LOQ	3.4	< LOQ	12.4	< LOQ	< LOQ	15.8 (28%)	20.3 (36%)	64%	13.8	0.1	13.7
Silty sand 2	52.7		< LOQ	3.8	< LOQ	11.0	< LOQ	< LOQ	14.8 (28%)	22.4 (43%)	71%	12.7	0.1	12.6
Silty sand 3	54.6		< LOQ	2.3	< LOQ	6.5	< LOQ	< LOQ	8.7 (16%)	17.7 (32%)	48%	13.2	0.2	13.0

‘LOQ’, limit of quantification; for the enzymatic extracts: SeMet, 2.0 mg Se kg^−1^; MetSeCys, 4.0 mg Se kg^−1^; SeVI, 0.8 mg Se kg^−1^; for the water extracts: SeMet, 1.0 mg Se kg^−1^; MetSeCys, 2.0 mg Se kg^−1^; SeVI, 0.4 mg Se kg^−1^. Different uppercase letters indicate significant (*P*-value < 0.05) differences

Elemental selenium represented an abundant contributing selenospecies, ranging 28–43% of the total selenium content (Table 1). This form of selenium was practically neglected for decades in selenium speciation, although it is often reported to be one of the major forms of selenium in selenized plants (Mounicou et al. 2009; Aborode et al. 2015; Both et al. 2018; Reynolds et al. 2020), including selenium accumulator species (*Astragalus bisulcatus*; Valdez Barillas et al. 2012) and the non-accumulator wheat (*Triticum aestivum*) as well (Xiao et al. 2021; Subirana et al. 2023).

However, turning the soil-derived selenospecies, selenate and selenite, into elemental selenium requires a huge reduction capacity from the plant; storing selenium in the form of nano- or microscale Se^0^ particles can be a part of detoxification, as it prevents excess selenium from entering into plant metabolism and provoking protein misfunctioning when no other efficient selenium removal pathways exist (e.g., accumulating non-proteinaceous selenoamino acids such as methylselenocysteine, selenolanthionine, selenocystathionine, and selenohomolanthionine) (White 2018). Indeed, the accumulation of elemental selenium in the root might be an explanation for the observation of Wang et al. (2023) on the restricted transport of selenium from the roots toward the stem and leaves of white cabbage. As highlighted by Pilon-Smits and LeDuc (2009), elemental Se might interfere with iron–sulfur cluster formation in the chloroplast and thus reduce the Se tolerance of the plant—but if excess Se is stored in the root section, the photosynthetic activity of the plants would not be hampered by this microelement.

On the other hand, Se^0^ accumulation in the root can also be the result of Se^0^-producing endosymbionts (Lindblom et al. 2013). When focusing on the *B. oleracea*-specific beneficial endophytic microorganisms (Card et al. 2015), *Bacillus amyloliquefaciens* (Liu et al. 2023), *Bacillus pumilus*, and *Bacillus subtilis* (Ikram and Faisal 2010) have all been proved to be able to reduce anionic inorganic selenium species into Se^0^. This observation indicates that elemental Se deposition in the root section cannot be regarded solely as a possible detoxification process driven by the plant but also as a natural bacterial symbiotic activity.

**Fig. 1:**
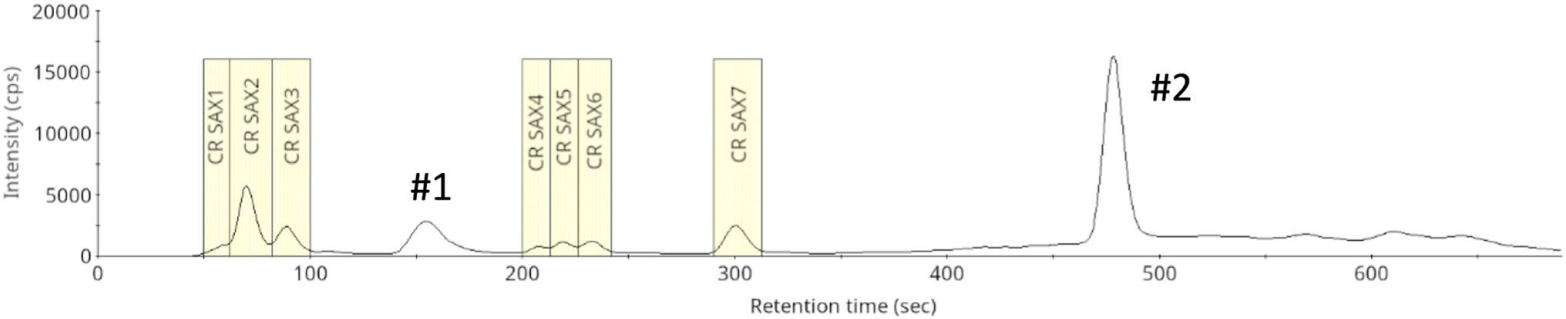
HPLC(SAX)–ICP-MS chromatogram of the concentrated water extract of cabbage root for the purpose of fraction collection, recorded on the ^78^Se isotope. Yellow squares mark the retention windows of the fractions.’#1’ denotes to selenomethionine, while #2 denotes to selenate

Concerning the two-step extraction process, inorganic selenate was the only water-soluble selenospecies that could be selectively quantified from the water extracts, and it made up 4–24% of total selenium content in the roots (Table 1, Fig. 1, Supplementary Fig. S1). Accordingly, the presence of selenate indicates incomplete biotransformation. The water extracts obtained from the root samples of the three different soil types did not differ considerably. Selenomethionine was present only under the limit of quantification, and there were approximately seven partly unresolved peaks that could not be unambiguously matched with the available selenium standards. The total selenium concentration of these peaks was estimated (Supplementary Fig. S2) and their summarized concentration covered approximately 2.1–3.2 mg Se kg^−1^ DW, which accounted for 3.5–6.1% of the total selenium content of the roots.

Enzymatic extraction complemented the first extraction step through the determination of selenomethionine, adding an additional 12–26% of total selenium recovery. The source of selenomethionine is the protein content of the roots that extends approximately between 8 and 20 g kg^−1^ DW (Table 1). Methylselenocysteine, one of the primary compounds synthesized for selenium detoxification in plants, was present in some of the extracts over the detection limit, but it could not be quantified as its concentration never reached the limit of quantification (LOQ). This observation matches the report of Mechora et al. (2012), where this selenocompound was also detected under the relevant LOQ of the analytical setup.

Without taking into account elemental Se and the unassigned water-soluble selenocompounds, the overall species recovery ranged between 16 and 50% (expressed in total selenium content) with the median of 28%. When completed with elemental Se, the recovery ranged between 48 and 87%, which is higher in the case of all samples than the recovery reported by Mechora et al. (2012). Therefore, involving Se^0^ quantification in the selenium speciation of plants seems to be an inevitable task to achieve high species recovery. However, there was still a proportion of selenium that could not be identified in the root extracts. Therefore, orthogonal LC-based purification was carried out to provide adequate concentration and purity of the unknown analytes for the electrospray ionization high-resolution mass spectrometry (ESI–HR-MS) analysis.

### Assignment and tentative identification of selenometabolites after two-dimensional orthogonal chromatographic purification

The application of strong anion (SAX) and strong cation (SCX) exchange chromatography in the purification processes intended for selenometabolite assignment by ESI–HR-MS setups has been reported for decades and can be considered a robust approach (McSheehy et al. 2002; do Nascimento da Silva et al. 2017; Both et al. 2018). In our case, the pooled and concentrated water extract of cabbage root samples was first subjected to SAX chromatography, which resulted in the detection of seven fractions before the elution of selenomethionine and between the elution of selenomethionine and selenate. The seven fractions obtained through the repeated fraction collection process were then subjected to SCX separation that finally allowed for the collection of 14 fractions (Supplementary Fig. S3).

Finally, the screening for selenocompounds in the 14 fractions obtained after the two-dimensional purification resulted in the detection of altogether eight compounds from four fractions, including isomers (Table 2). This low ratio of species recovery can be explained by several reasons. First, the implementation of a parallel LC–ICP-MS/ESI–MS detection where the selenium-specific ICP-MS traces (peaks) directly allocate the narrow retention time frames of selenocompounds in the ESI–MS full-scan spectra requires a complex analytical setup where the applied LC parameters (especially the eluents) are fully compatible with both mass spectrometers—such setups are relatively few in number. Second, ESI–MS-related issues (e.g., species decomposition in the ion source, matrix suppression, isobaric interferences even in the case of applying high-resolution mass spectrometers, limited desktop access to the use of both ionization modes, to orthogonal/HILIC vs. RP/separation systems, and to vendor-specific software tools) clearly hamper the high assignment rate of selenospecies. Where the cationic fractions were eluted close to the void volume of the chromatographic setup, or the concentration of selenium in the given fraction was too low, the load from the co-eluting matrix constituents inherently hampered the successful detection of the selenium pattern in the relevant fraction. Third, species decomposition can also occur during the purification steps where repetitive dissolution and drying (evaporation or freeze-drying) procedures are addressed.

**Table 2:** Liquid chromatography–high-resolution mass spectrometry data of the Se-enriched cabbage root selenometabolites detected in the study

Sign	RT #1, min	RT #2, min	Ionization	Suggested elemental composition (neutral)	Name, note	Theoretical *m/z* on ^80^Se	Experimental *m/z* on ^80^Se	Δ, ppm	Structure code	Figure
SCX1-SAX3	**1.55**	0.78	[M + H]^+^	C_14_H_25_NO_3_Se	Possibly with an end-chain R-Se-CH_3_ moiety	336.10724	336.10701	− 0.68	-	Supplementary Figs. S4, S5
SCX1-SAX6	**0.61**	0.62	[M + H]^+^	C_8_H_14_O_6_Se	Doubly deaminated hydroxy-selenohomolanthionine	287.00284	287.00256	− 0.98	Se-01	Figure 2
SCX1-SAX7	**2.26**	1.42	[M + H]^+^	C_9_H_16_O_5_Se	–	285.02357	285.02373	0.56	Se-05	Figure 7, Supplementary Figs. S11, S12
	0.62	**0.60**	[M + H]^+^	C_8_H_13_NO_5_Se	Mono-deaminated selenohomolanthionine, oxidized into a ketone (deamino-2-oxo-selenohomolanthionine) or into an aldehyde	284.00317	284.00291	− 0.92	Se-03	Figures 4, 5a, 6; Suppl. Fig. S7a, S8
	0.96	**0.67**				284.00317	284.00291	− 0.92		Figures 4, 5b, 6; Supplementary Fig. S7b, S8
	1.00	**0.79**				284.00317	284.00358	1.44		Figures 4, 5c, 6; Supplementary Fig. S7c, S8
	**0.91**	0.75	[M-H]^−^	C_8_H_15_NO_5_Se	Mono-deaminated hydroxy-selenohomolanthionine	284.00427	284.00394	− 1.16	Se-02	Figure 3b, Supplementary Fig. S6b
			[M + H]^+^			286.01882	286.01912	1.05		Figure 3a, Supplementary Fig. S6a
SCX2-SAX2	**1.86**	1.07	[M + H]^+^	C_9_H_18_O_5_Se	–	287.03922	287.03941	0.66	Se-04	Figure 8, Supplementary Figs. S9, S10

Retention time values in bold indicate which gradient was used during the MS/MS analysis

In the first fraction collected from the SCX purification step, seven selenocompounds from three SAX fractions (#3, #6, and #7) could be detected. A formerly unreported species at *m/z* 336.10701 with the only possible elemental composition of C_14_H_25_NO_3_Se (neutral state; within ≤ 2 ppm mass accuracy) was detected (Suppl. Fig. S4) in fraction #3. Unfortunately, no characteristic selenized fragments could be identified in the MS/MS spectrum (Suppl. Fig. S5). The molecule might be a selenoamino acid derivative, for example, a selenohomocysteine-derived species according to its elemental composition, that is, carrying one nitrogen and ≥ 2 oxygen atoms, but the lack of selenium-containing fragments (except for two water losses; *m/z* 336—> *m/z* 318—> *m/z* 300) and characteristic amino acid-derived losses (*m/z* 46 for formic acid and *m/z* 17 for ammonia) excludes this option. Indeed, this molecule can contain an end-chain R-Se-CH_3_ moiety that leaves during fragmentation without resulting in any selenized fragment in the positive ion mode. Taking into account that the compound was not detected in the negative ion mode and no other characteristic information could be gained, no structure can be proposed for this metabolite.

**Fig. 2:**
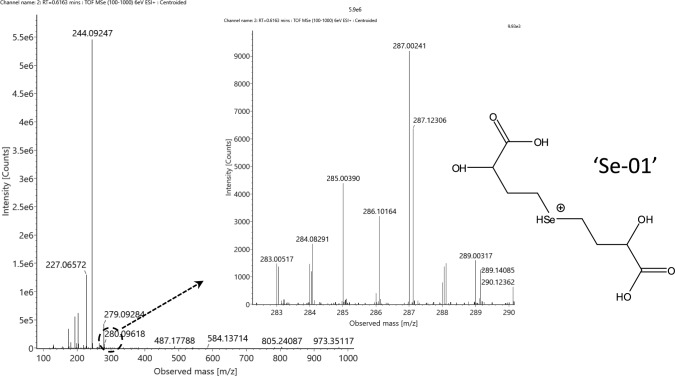
Full-scan spectrum of the tentative C_8_H_14_O_6_Se selenocompound (neutral composition), detected in the ESI’ + ’ mode at RT = 0.61 min (Table 2). The insets show the magnified spectrum of the monoselenized species, together with the suggested structure of the compound (’Se-01’), doubly deaminated hydroxy-selenohomolanthionine

In the fraction #6, a selenium pattern-containing species was detected at *m/z* 287.00256 (Fig. 2). The molecule showed a considerable isobaric interference during both gradient elution modes on the monoisotopic (^80^Se) isotopologue, which hampered its selective MS/MS fragmentation, while the intensity of the ^78^Se isotopologue was too low to provide an adequate signal-to-noise ratio for MS/MS analysis. However its structural identification is not possible at this stage, its elemental composition (C_8_H_14_O_6_Se; neutral state) is in agreement with a synthetic by-product detected during the preparation of deamino-hydroxy-analog of selenomethionine, *R*,*S*−2-hydroxy-4-methylselenobutanoic acid (HMSeBA; EFSA 2013). Accordingly, a symmetric structure originating from the demethylation and coupling of two HMSeBA fragments can be tentatively suggested, as presented in Fig. 2 (structure ‘Se-01’). Indeed, this molecule can also be regarded as doubly deaminated selenohomolanthionine that is oxidized into the hydroxyl forms at both moieties of the molecule.

**Fig. 3:**
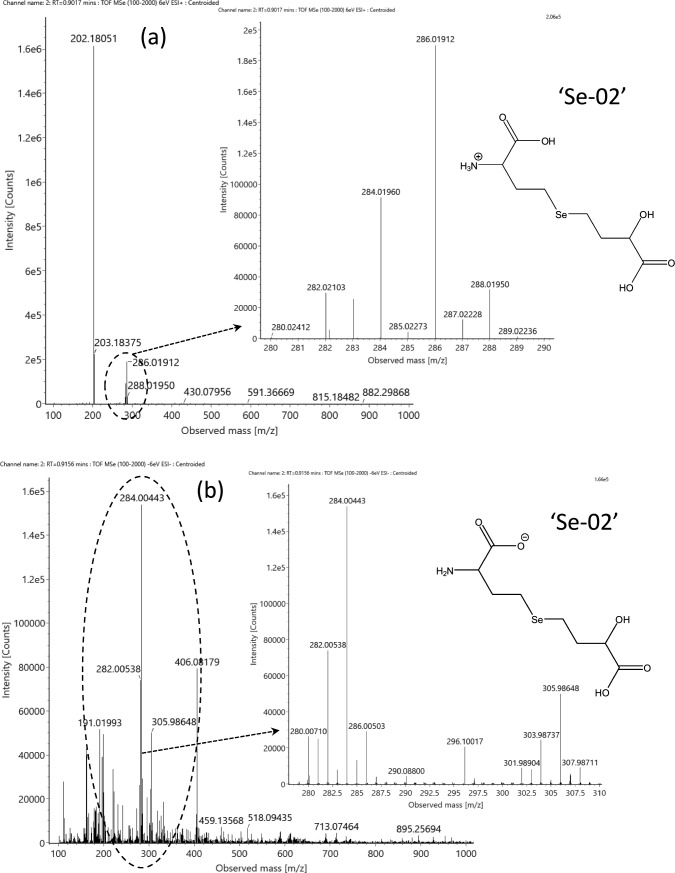
Full-scan spectrum of mono-deaminated hydroxy-selenohomolanthionine, detected at RT = 0.91 min (Table 2). The insets show the magnified spectrum **a** In the ESI’ + ’ mode, **b** In the ESI’–’ mode (together with its sodium adduct). Please note that ionization can occur at different moieties of the molecule; therefore, the actual version (’Se-02’) serves only for presentation purposes. For the MS/MS spectra, see Suppl. Fig. S6

The presence of deamino-hydroxy-moieties is supported by the detection of a molecule in the fraction #7 at *m/z* 286.01912 (ESI ‘ + ’; *m/z* 284.00394 in the ESI ‘- ‘ mode) that corresponds to the neutral composition of C_8_H_15_NO_5_Se (Fig. 3a, b). The MS/MS spectra (Suppl. Fig. S6 a, b) acquired in both ionization modes indicate this molecule is mono-deaminated hydroxy-selenohomolanthionine, first reported by Ouerdane et al. (2013) in mustard seeds (*Brassica nigra*) and later in garlic (*Allium sativum*) by Ruszczyńska et al. (2017) and in alfalfa by Domokos-Szabolcsy et al. (2024). Accordingly, the further deamination of this molecule can be the origin of the *m/z* 287.00256 metabolite (‘Se-02’) detected in fraction #3.

**Fig. 4:**
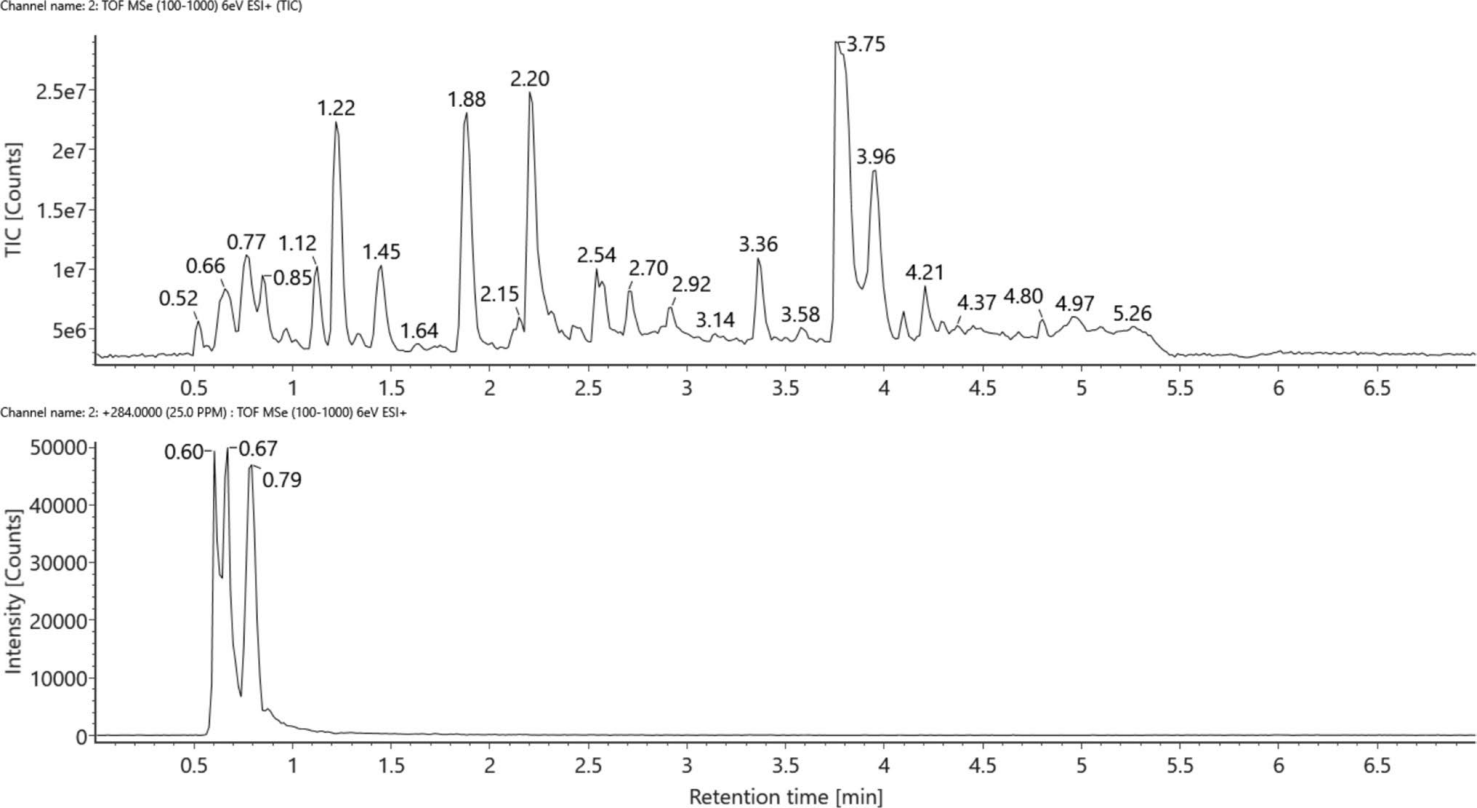
The total ion chromatogram (TIC) of the SCX1–SAX7 fraction and the extracted ion chromatogram (EIC) of *m/z* 284.00. The retention time values of the C_8_H_13_NO_5_Se species (theoretical *m/z* 284.00317 as C_8_H_14_NO_5_Se^+^) are as follows: RT = 0.60 min, 0.67 min, and 0.79 min

**Fig. 5:**
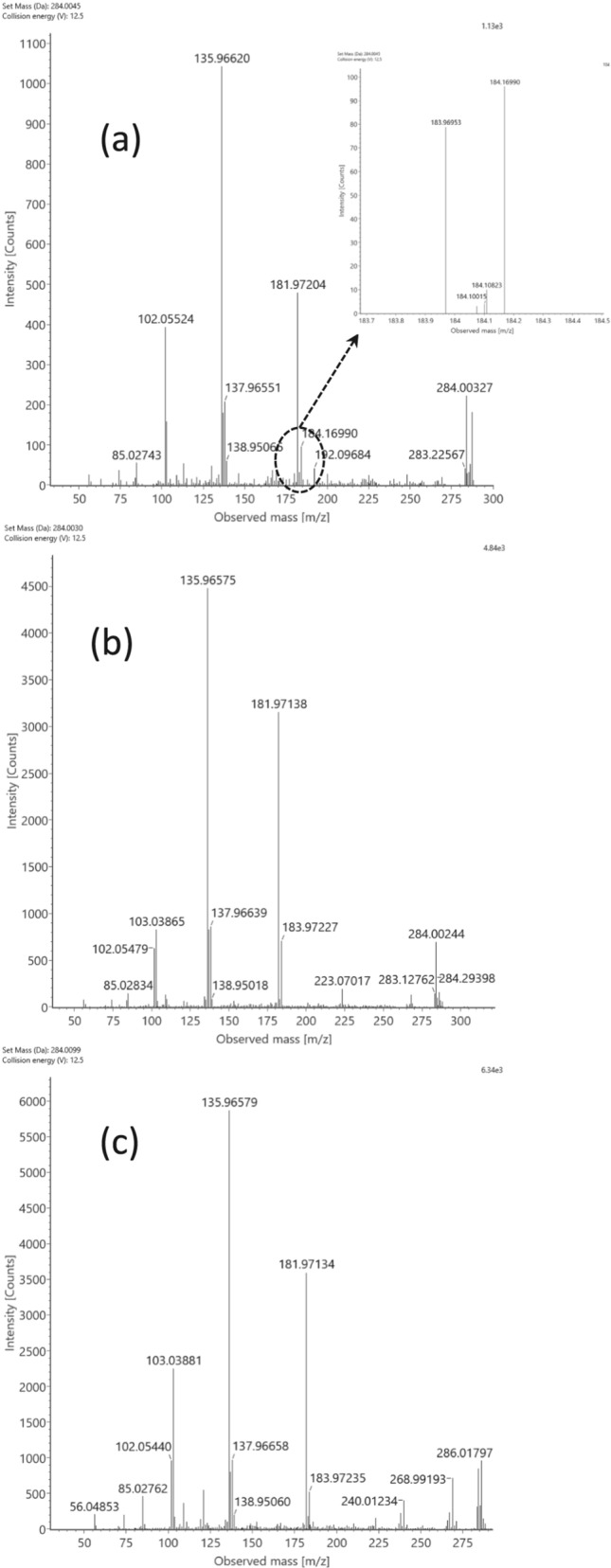
MS/MS spectra of the C_8_H_13_NO_5_Se (neutral state; theoretical *m/z* 284.00317 for C_8_H_14_NO_5_Se^+^) selenocompound detected in the ESI’ + ’ mode. **a** At RT = 0.60 min, the insert shows the adjacent fragments at *m/z* 184. **b** At RT = 0.67 min. **c** At RT = 0.79 min

Suggesting the detection of selenohomolanthionine derivatives is supported by the detection of a set of three selenocompound isomers in fraction #7 at m/z 284.00291 and 284.00358 (ESI ‘ + ’; Table 2). This molecule possesses the elemental composition of C_8_H_13_NO_5_Se (neutral state), which was recently reported as a single species in selenized Torula yeast (Bierła et al. 2017) and in alfalfa (Domokos‑Szabolcsy et al. 2024), with the tentative origin from selenohomolanthionine after its possible oxidative mono-deamination to ketone (R_2_HC-NH_2_→ R_2_C = O; Δ = − 1.032 Da) to form deamino-2-oxo-selenohomolanthionine. On the other hand, the extracted ion chromatogram of the theoretical mass (*m/z* 284.00317) shows at least three isomers at slightly different retention times (Fig. 4, Supplementary Fig. S7a, b, c). The abundance of the three main peaks was high enough to record MS/MS spectra (Fig. 5a, b, c) that all contain the two characteristic fragments of selenohomocysteine (*m/z* 135.96678 /C_3_H_6_NSe^+^/; 181.97267 /C_4_H_8_NO_2_Se^+^/). Of note is the lack of the selenohomolanthionine-specific and the mono-deaminated hydroxy-selenohomolanthionine-specific fragments (*m/z* 166.9606 /C_4_H_7_O_2_Se^+^/; 183.9871 /C_4_H_10_NO_2_Se^+^/. Besides, there are three fragments that show different ratio in the function of retention time, that is, the more hydrophobic properties the compound bears on the RP column, (i) the higher is the *m/z* 103.038 *vs*. *m/z* 102.055 ratio (C_4_H_7_O_3_^+^ *vs.* C_4_H_8_NO_2_^+^, respectively), and (ii) the higher the abundance of the selenized fragments at m/z 120.956 (C_3_H_5_Se^+^), 240.01234 (C_7_H_14_NO_3_Se^+^, possibly due to an usual CO_2_ loss from the oxygen-rich moiety of the molecule) and 268.99193 (C_8_H_13_O_5_Se^+^; supposed fragment rearrangement product).

**Fig. 6:**
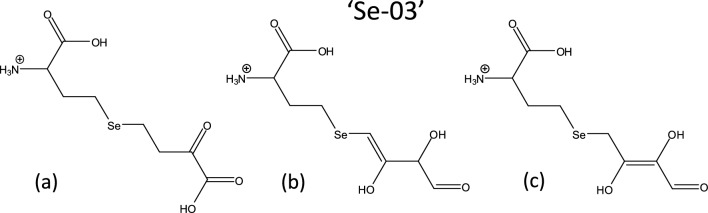
Structures (grouped as’Se-03’) suggested for the C_8_H_14_NO_5_Se^+^ selenospecies. **a** Deamino-2-oxo-selenohomolanthionine. **b**, **c** Two tentative structures of the aldehyde forms

A possible explanation of the different ion abundances and the different retention times shedding light on more hydrophobic selenospecies might be the presence of an aldehyde group instead of the carboxylic group. Accordingly, tentative structures (grouped as ‘Se-03’) are suggested in Fig. 6: subfigure (a) presents deamino-2-oxo-selenohomolanthionine, while subfigures (b, c) show the tentative structures of the aldehyde forms, eluting later in the chromatogram. It is to note that no −18 Da water loss events were detected during the MS/MS fragmentation of the isomers (Suppl. Fig. S8); the possible reason behind this might be the keto-enol tautomeric behavior of hydroxyl groups in this unsaturated moiety of the molecule.

**Fig. 7:**
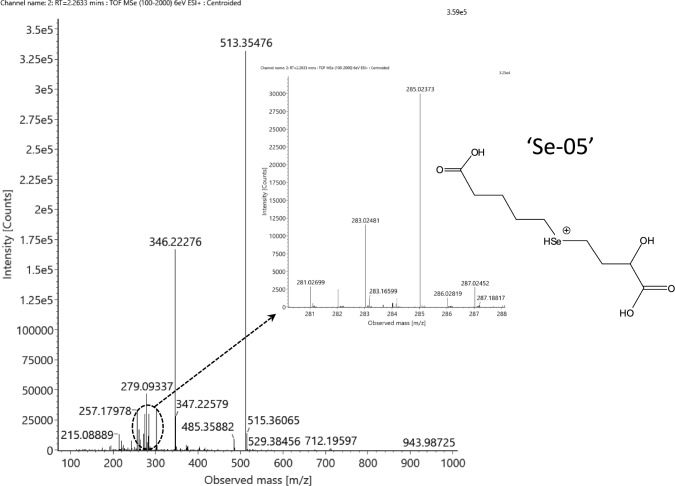
Full-scan spectrum of the tentative C_9_H_16_O_5_Se selenocompound (neutral state) detected in the ESI’ + ’ mode at RT = 2.26 min. The inset shows the magnified spectra of the monoselenized species and a tentative structure (’Se-05’)

**Fig. 8:**
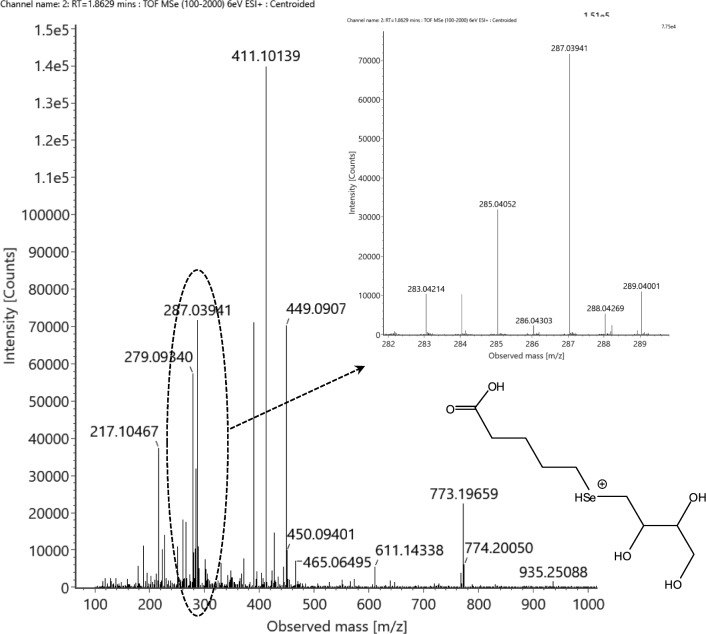
Full-scan spectrum of the C_9_H_18_O_5_Se selenocompound (neutral state) detected in the ESI’ + ’ mode at RT = 1.86 min. The inset shows the magnified spectra of the monoselenized species and a tentative structure (’Se-04’)

In the same fraction (#7), there was one more selenocompound detected at m/z 285.02373 that corresponds to the neutral elemental composition of C_9_H_16_O_5_Se within 2 ppm mass accuracy (Fig. 7). Besides, another selenocompound could also be assigned in the SCX2-SAX2 fraction at m/z 287.03941 that matches the neutral element composition of C_9_H_18_O_5_Se (Fig. 8). No selenocompound has been previously reported that contains nine carbon atoms without any nitrogen atom in its structure.

Concerning the MS/MS spectra of the C_9_H_19_O_5_Se^+^ molecule, they featured a long series of selenium-containing fragments (Supplementary Figs. S9 a, b). To achieve a fragment-rich MS/MS spectrum, a considerably high collision energy was also required, which can be associated with the high degree of saturation of the molecule. However, the structural evaluation of the compound is anyhow limited: (i) while five oxygen atoms are present, neither water (− 18 Da) nor direct formic acid losses (− 46 Da) are detected that would help allocate oxygens in the molecule; (ii) only one selenized fragment (one out of seven) contains an oxygen atom, which still refers to the presence of functional groups ready to leave during MS/MS fragmentation; (iii) assignment of the selenium-free fragments is unclear due to the possible fragment rearrangement events that might occur due to the suspected internal selenoether structure. Indeed, the allocation of the only one double bond is the key to suggesting a tentative structure: either it is present as a carbon–carbon double bond, or as a keto group, or as a part of a carboxylic group. Taking into account that the highest number of carbon atoms (four) is detected in an oxygen-free selenized fragment *m/z* 134.97, it might be suggested that this fragment originates from the break of the internal selenoether bond and a subsequent formic acid loss. Accordingly, the double bond might be in the form of a carboxylic group, and the leftover three oxygen atoms should be arranged as hydroxyl groups on the other side of the selenoether moiety. This tentative structure (‘Se-04’), together with the suggested selenium-containing fragments, is presented in Fig. 8 and Supplementary Fig. S10.

Concerning the C_9_H_17_O_5_Se^+^ molecule, there are several fragments that should be highlighted during the structural elucidation. First, the presence of the selenized fragments *m/z* 132.95, 134.97 and 164.98 also refers to a mid-chain (internal) selenoether and not to an end-chain R-Se-CH_3_ species (Supplementary Fig. S11). The first two fragments show matching with the MS/MS fragmentation pattern of the C_9_H_19_O_5_Se^+^ molecule, but the last selenized fragment and the selenium-free fragments (*m/z* 57.034, 85.028, and 103.039) are different. These three latter fragments were although characteristic of mono-deaminated hydroxyl-selenohomolanthionine (Suppl. Fig. S6 a); therefore, there is a hint about the structure of the compound (‘Se-05’), that is, the mono-deaminated moiety of selenohomolanthionine is attached to a C_5_-alkyl chain, possibly with a carboxylic group, similarly to the C_9_H_19_O_5_Se^+^ species (Fig. 7 and Supplementary Fig. S12). The reason behind suggesting a carboxylic group instead of proposing a keto (or aldehyde) together with a hydroxyl group is the higher tendency to leave in the form of a formic acid loss, thus creating oxygen-free fragments with four carbon atoms in the fragments. Clearly, it cannot be declared in either of the two structures of ‘Se-04’ and ‘Se-05’ whether the alkyl chain is branched or not. Accordingly, both structures are tentative only.

After assigning several derivatives (including formerly reported and tentative ones as well) of selenohomolanthionine, we conducted a directed search for this selenospecies in the 14–14 full-scan spectra (14 fractions in two ionization modes), without any matches. Indeed, two-headed amino acids like selenohomolanthionine or selenocystathionine can be retained on dedicated ion-pairing reversed phase or HILIC setups—accordingly, the presence of selenohomolanthionine in the cabbage root fractions cannot be theoretically excluded.

## Conclusions

In terms of selenium species recovery, water and proteolytic extractions of the fiber-rich structure of cabbage root could provide approximately one-third of the total selenium content, revealing the presence of selenate, selenomethionine, and the tentative derivatives of selenohomolanthionine. While the detection of selenomethionine clearly indicates the biotransformation of selenate by the plant and its incorporation into plant proteins, the origin of the water-insoluble Se^0^ and the water-soluble and non-proteinaceous low-molecular-weight selenocompounds cannot be unambiguously linked to the plant selenium metabolism. Indeed, the microbiome-related activity of the soil–root system, especially in the light of the high elemental Se concentration detected in all samples, can also be the origin of the selenocompounds, taking into account that selenohomolanthionine (Bierła et al. 2017) and other selenocompounds (e.g., γ-Glu-selenomethionine, Zhu et al. 2022) have all been recently identified as major microbial selenometabolites. This observation calls attention to novel research policies where the plant–root–microbiome system should be analyzed holistically, and selenium speciation should be accompanied with metagenomics to unearth possible interactions between plant and microbial selenium metabolisms.

## Supplementary Information

Below is the link to the electronic supplementary material.

Supplementary file1 (PDF 1853 KB)

Supplementary file2 (PDF 713 KB)

## Data Availability

Data will be made available on request.

## Abbreviations

ESI: Electrospray ionization
HR: High resolution
ICP: Inductively coupled plasma
LC: Liquid chromatography
SAX: Strong anion exchange
SCX: Strong cation exchange
