# Supplementary material for: Assignment of low-molecular-weight selenometabolites in the root section of white cabbage
Source: Planta. 2025 Mar 1;261(4):71. doi: 10.1007/s00425-025-04651-y (PMC11872985; doi:10.1007/s00425-025-04651-y)
Supplement: Supplementary file 1 — Supplementary file1 (PDF 1853 KB) [file 425_2025_4651_MOESM1_ESM.pdf]

ELECTRONIC SUPPLEMENTARY MATERIAL

for the paper entitled

Assignment of low molecular weight selenometabolites in the root section of white cabbage

published in

Planta

(**DOI :** 10.1007/s00425-025-04651-y)

authored by

Áron Soós[1], Béla Kovács[1], Tünde Takács[2], Márk Rékási[2], Péter Dobosy[3], Csaba Szőke[4], Mihály Dernovics[5]*, Péter Ragályi[2]

[1]Institute of Food Science, Faculty of Agricultural and Food Sciences and Environmental Management, University of Debrecen, Böszörményi út 138, Debrecen, 4032, Hungary

[2]Institute for Soil Sciences, HUN-REN Centre for Agricultural Research, Fehérvári út 132-144, Budapest H-1116, Hungary

[3]Institute of Aquatic Ecology, HUN-REN Centre for Ecological Research, Karolina út 29, Budapest H-1113, Hungary

[4]Department of Maize Breeding, Agricultural Institute, HUN-REN Centre for Agricultural Research, Brunszvik u. 2, Martonvásár H-2462, Hungary

[5]Department of Plant Physiology and Metabolomics, Agricultural Institute, HUN-REN Centre for Agricultural Research, Brunszvik u. 2, Martonvásár H-2462, Hungary

*corresponding author's e-mail address: dernovics.mihaly@atk.hun-ren.hu

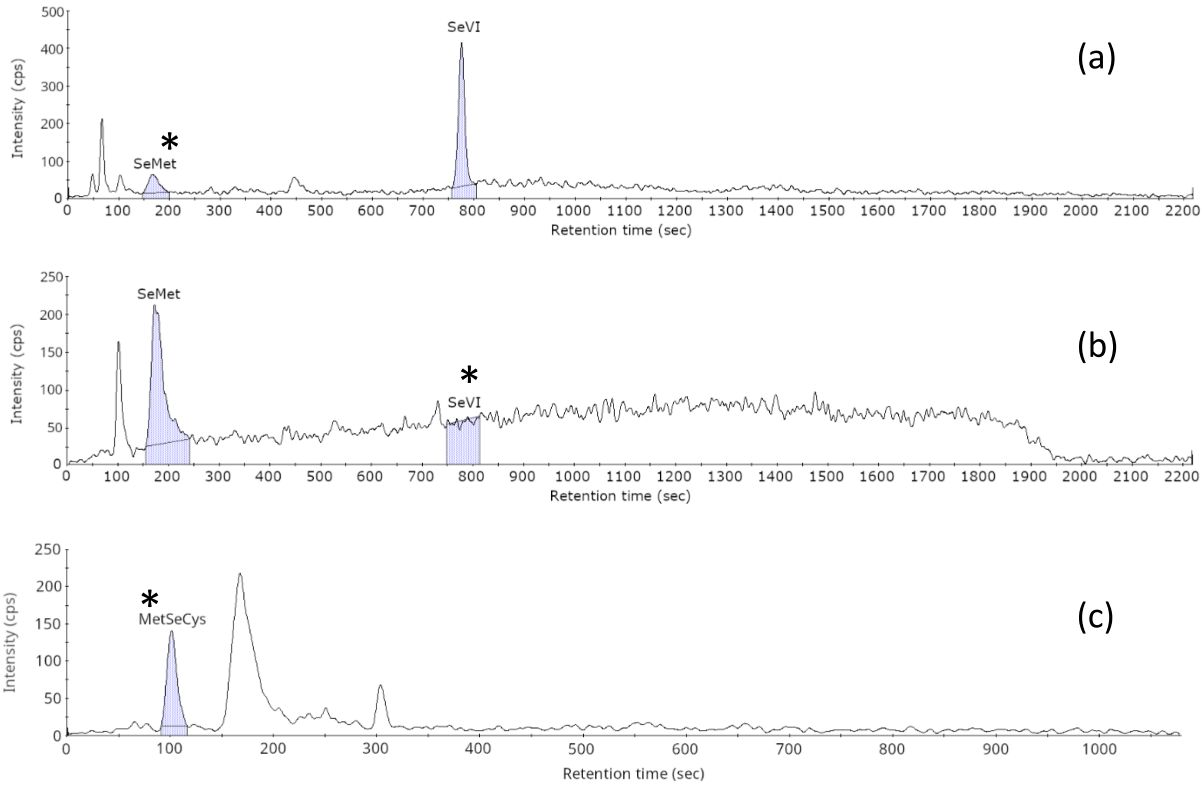

Suppl. Fig. S1 Typical HPLC(SAX)-ICP-MS chromatograms. **a** The water extract of cabbage root. **b** The enzymatic extract of cabbage root. **c** The enzymatic extract of cabbage root with a modified gradient program. All the chromatograms are recorded on the ^{78}Se isotope. '*' indicates the given species was detected under the actual limit of quantification.

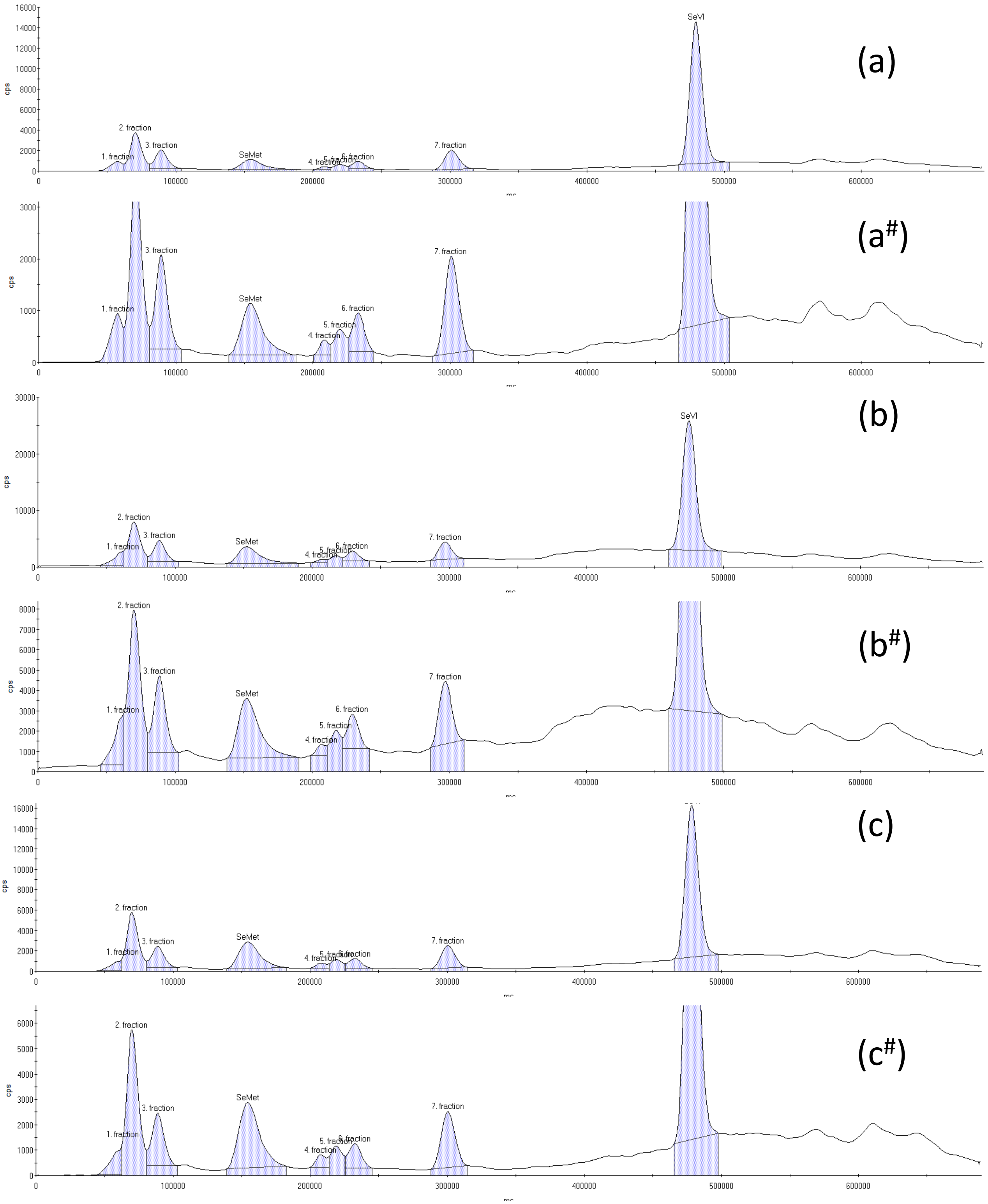

	mg kg^{-1} Se (estimated)							
Soil type/fraction No.	1	2	3	4	5	6	7	total Se
Sandy	0.19	0.77	0.37	0.05	0.14	0.15	0.45	**2.12**
Silt-Sand	0.26	1.29	0.55	0.06	0.29	0.25	0.48	**3.18**
Silty	0.20	1.17	0.43	0.09	0.23	0.20	0.51	**2.84**

Suppl. Fig. S2 SAX-ICP-MS chromatograms of the water extracts of cabbage root. **a** From sandy soil. **b** From silt-sand soil. **c** From silty soil. All the chromatograms are recorded on the ^{78}Se isotope. Chromatograms with '#' marks are the magnified versions of the original runs. The table lists the estimated total selenium contents of each fraction.

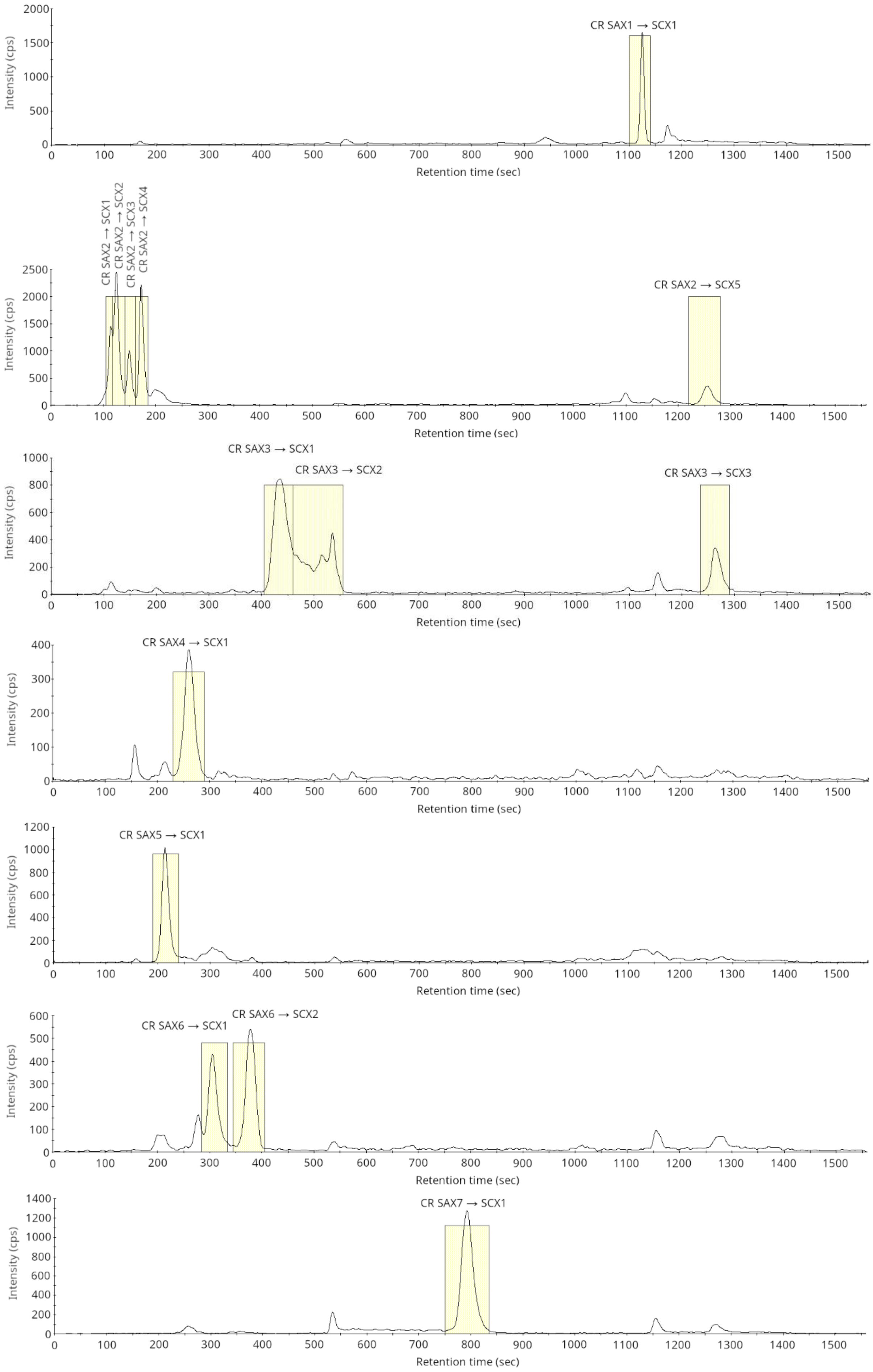

Suppl. Fig. S3 SCX-ICP-MS chromatograms of the SAX no. 1-7 fractions of cabbage root after SAX purification, recorded on the ^{78}Se isotope (see **Fig. 1** and **Suppl. Fig. S2**). Yellow squares mark the retention windows of the 14 fractions collected for the LC-ESI-HR-MS experiments.

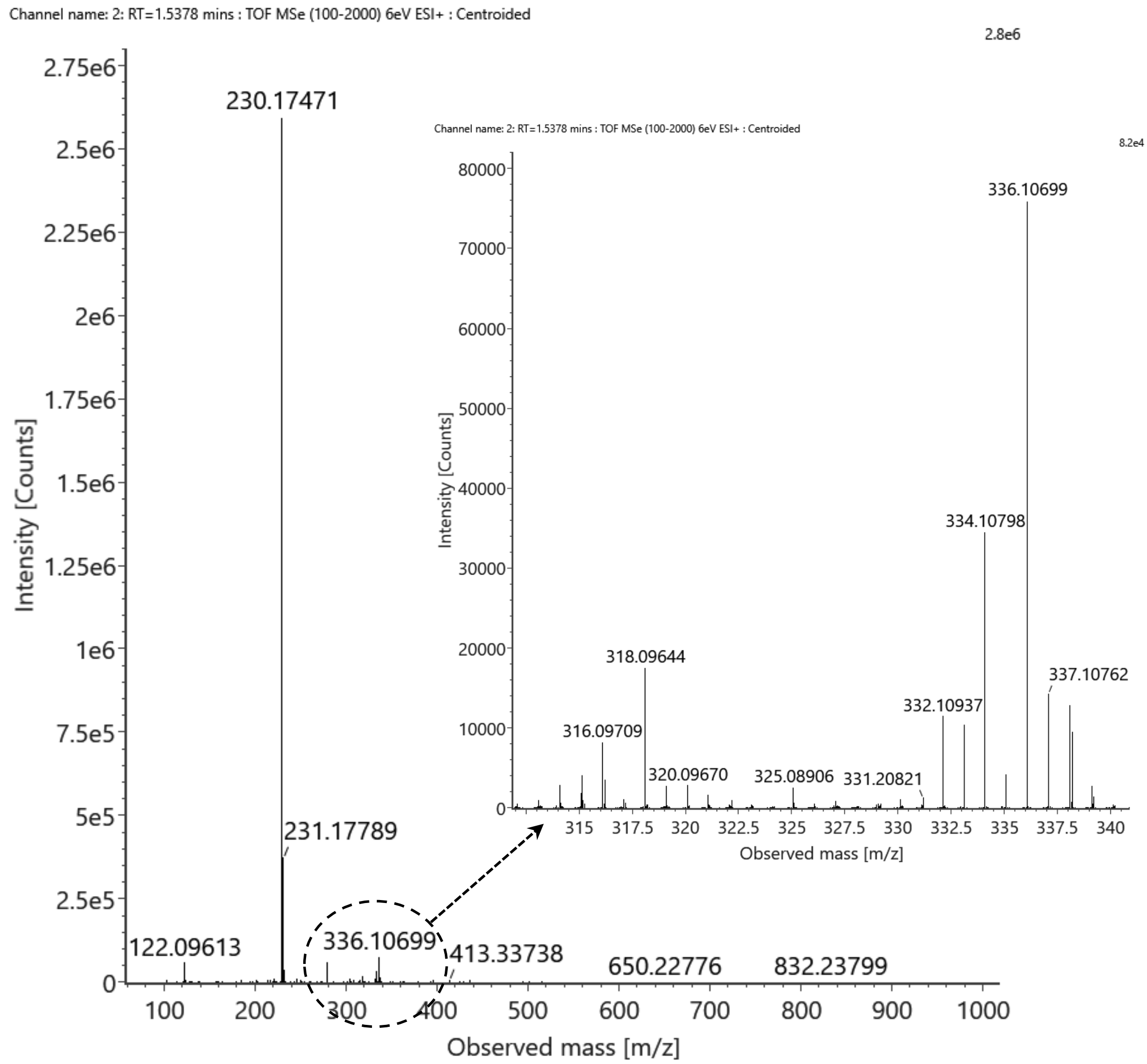

Suppl. Fig. S4 Full scan spectrum of the $C_{14}H_{25}NO_3Se$ selenocompound (neutral composition), detected in the ESI '+' mode at RT=1.55 min (Table 2). The inset shows the magnified spectrum of the monoselenized species, together with its ion source fragment (-18 Da; water loss).

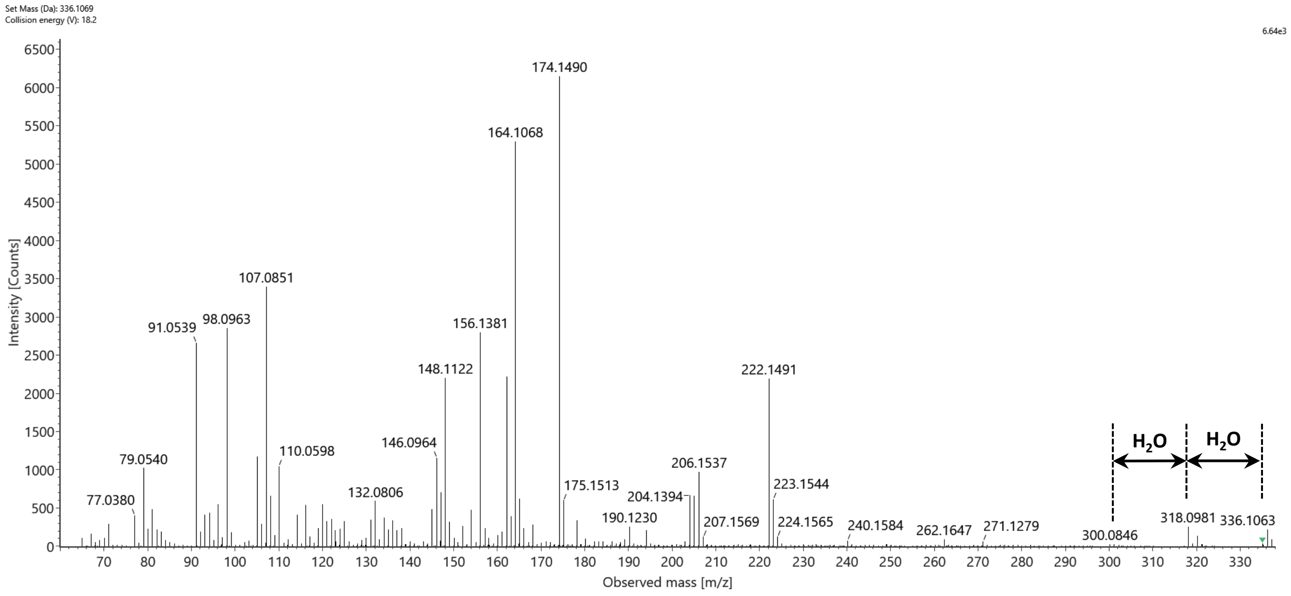

Suppl. Fig. S5 MS/MS spectrum of the $C_{14}H_{25}NO_3Se$ selenocompound (neutral composition), detected in the ESI '+' mode (collision energy: 18.2 eV).

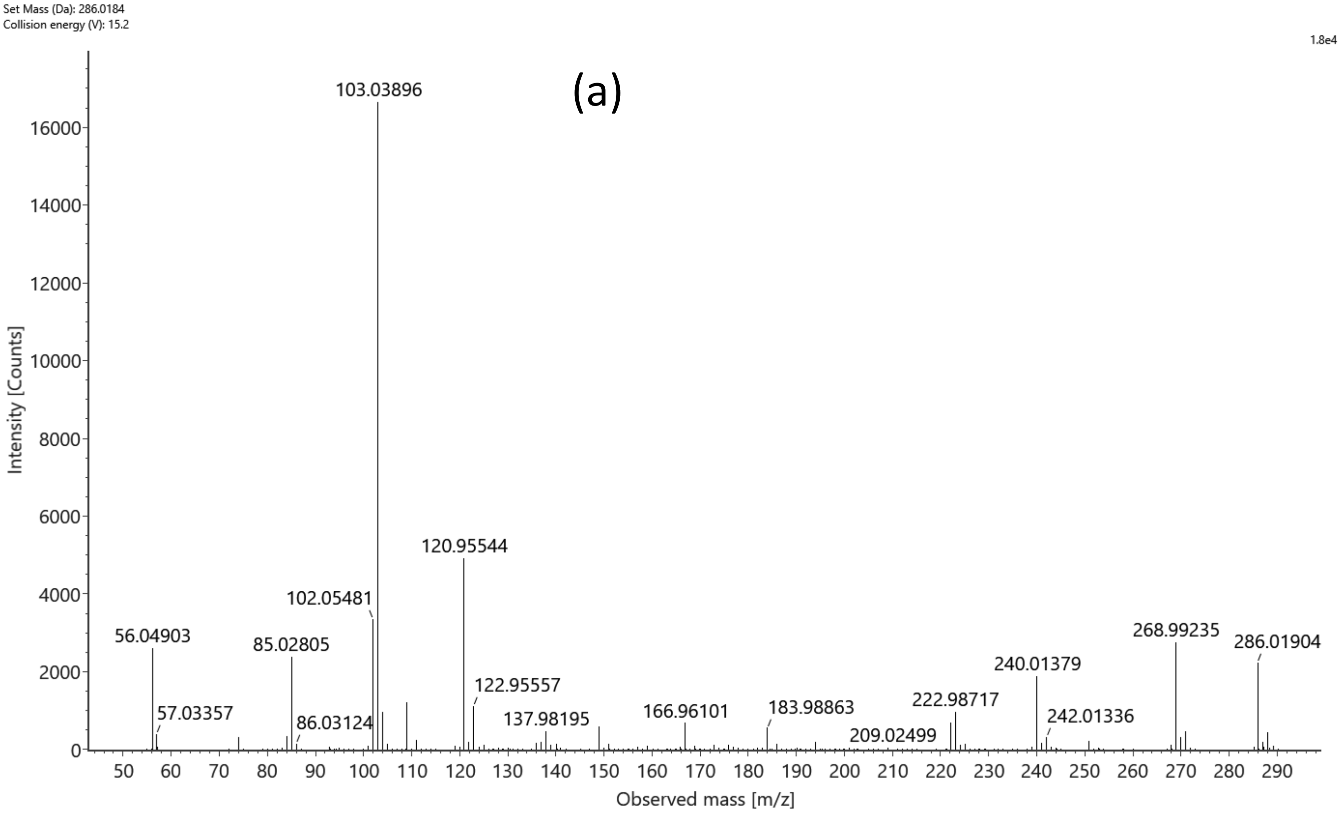

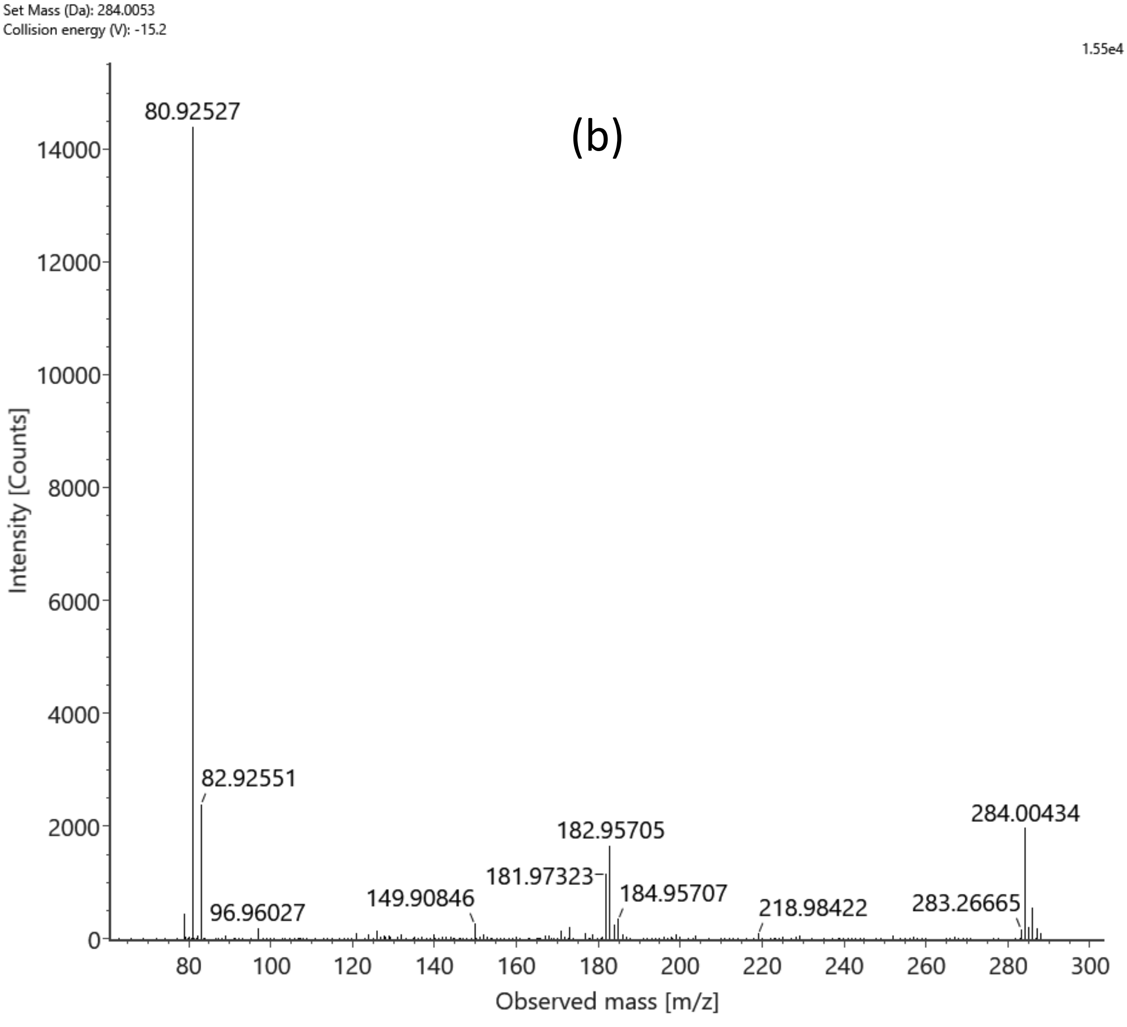

Suppl. Fig. S6 MS/MS spectra of mono-deaminated hydroxy-selenohomolanthionine. **a** Detected in the ESI ’+’ mode. **b** Detected in the ESI ’-’ mode.

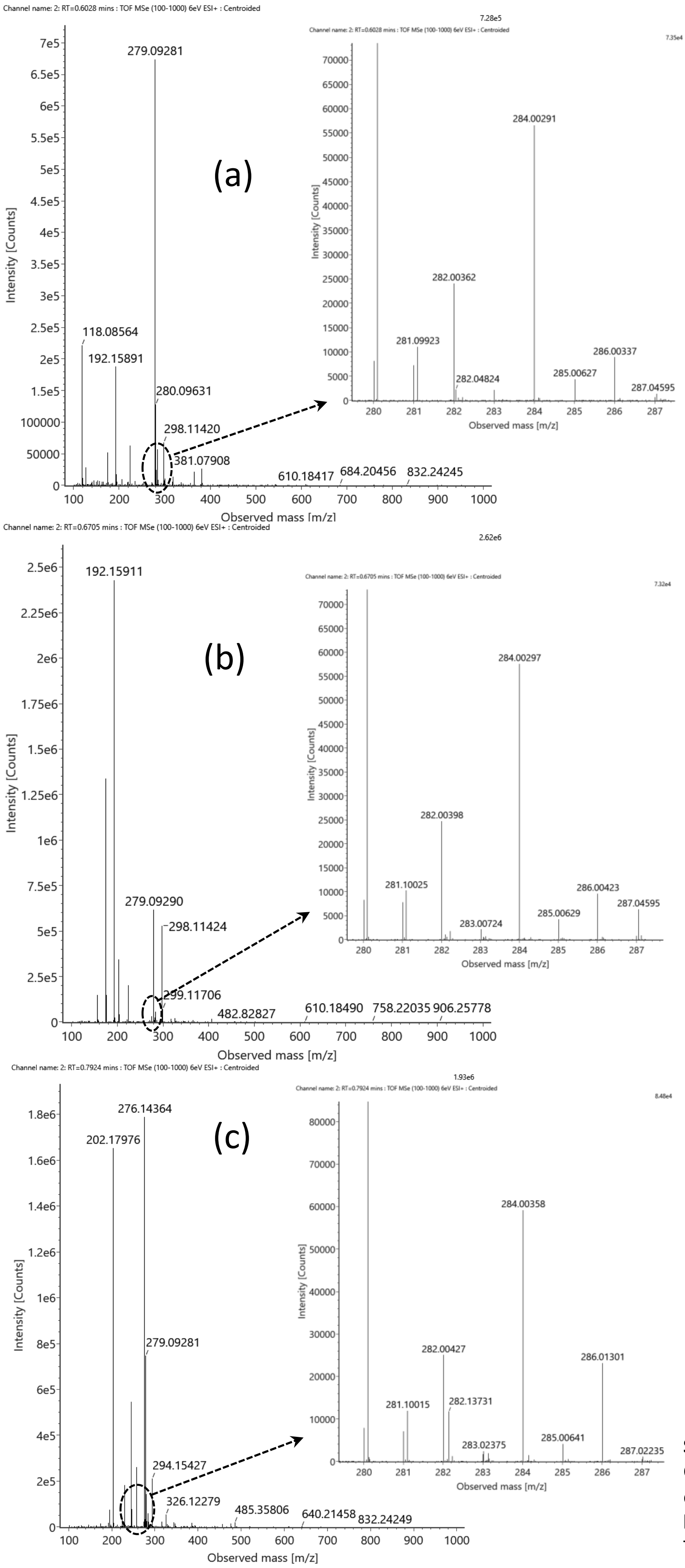

Suppl. Fig. S7 Full scan spectra of the $C_8H_{13}NO_5Se$ selenocompounds (neutral state) detected in the ESI '+' mode. **a** At RT=0.60 min. **b** At RT=0.67 min. **c** At RT=0.79 min (Table 2). The insets show the magnified spectra of the monoselenized species.

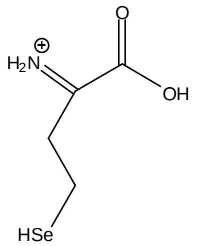

Chemical Formula: $C_4H_8NO_2Se^+$
Exact Mass: 181.9715

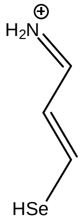

Chemical Formula: $C_3H_6NSe^+$
Exact Mass: 135.9660

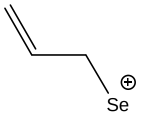

Chemical Formula: $C_3H_5Se^+$
Exact Mass: 120.9551

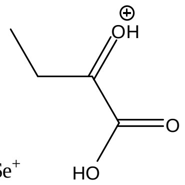

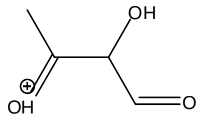

Chemical Formula: $C_4H_7O_3^+$
Exact Mass: 103.0390

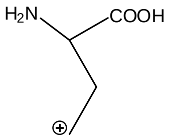

Chemical Formula: $C_4H_8NO_2^+$
Exact Mass: 102.0550

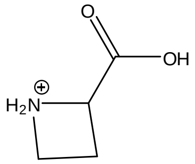

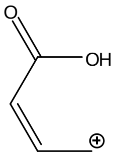

Chemical Formula: $C_4H_5O_2^+$
Exact Mass: 85.0284

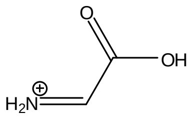

Chemical Formula: $C_2H_4NO_2^+$
Exact Mass: 74.0237

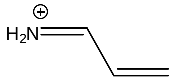

Chemical Formula: $C_3H_6N^+$
Exact Mass: 56.0495

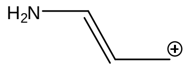

Suppl. Fig. S8 Tentative structures suggested for the fragments obtained from the MS/MS analysis of the isomers of the $C_8H_{14}NO_5Se^+$ selenospecies. Please note that the structures are subject to change, e.g., ionization and neutral losses can occur at different moieties, therefore the structures are intended for demonstrative purposes only.

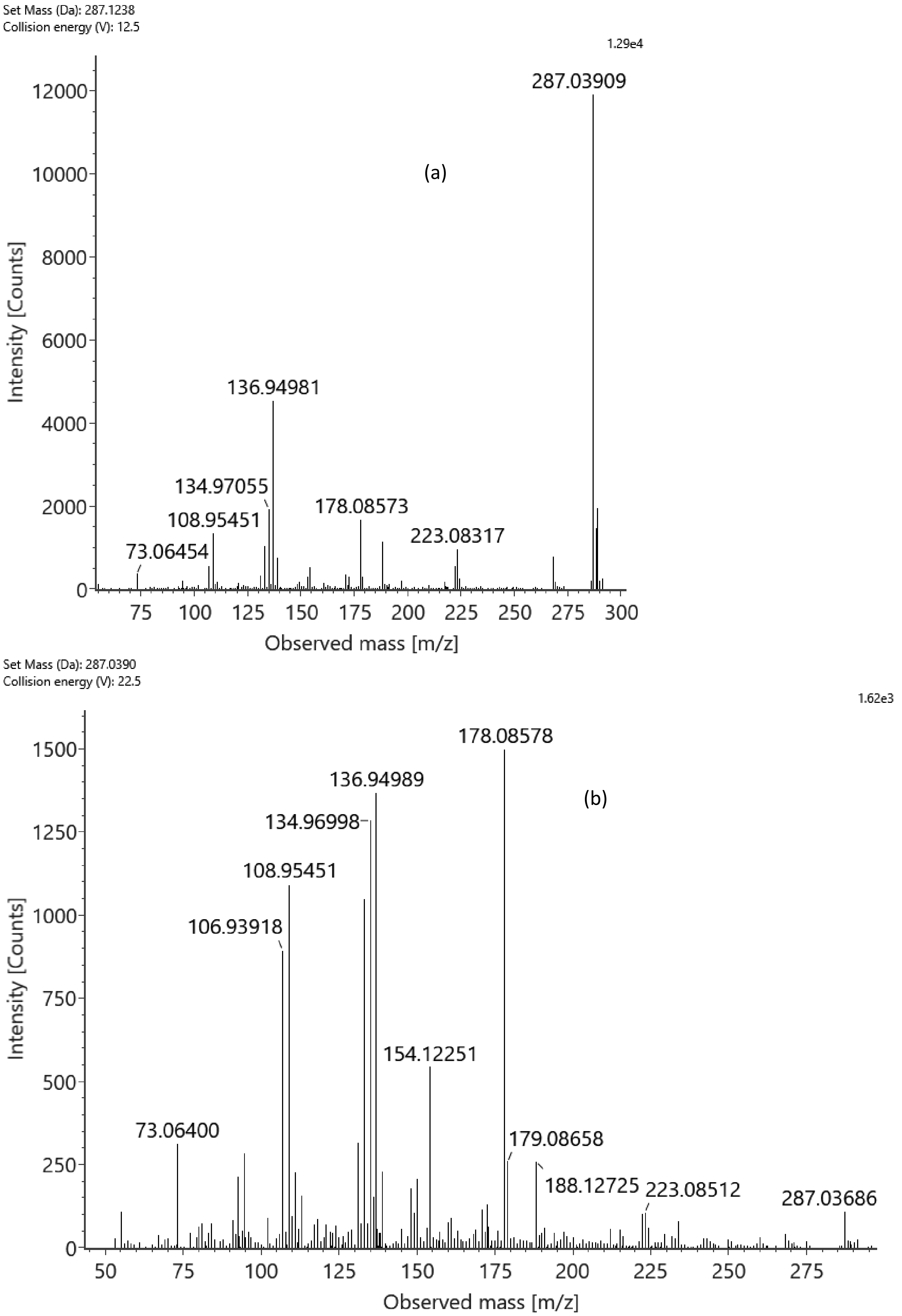

Suppl. Fig. S9 MS/MS spectra of the $C_9H_{18}O_5Se$ (neutral state; theoretical *m/z* 287.03922 for $C_9H_{19}O_5Se^+$) selenocompound recorded in the ESI '+' mode. **a** At 12.5 eV collision energy setting. **b** At 22.5 eV collision energy setting.

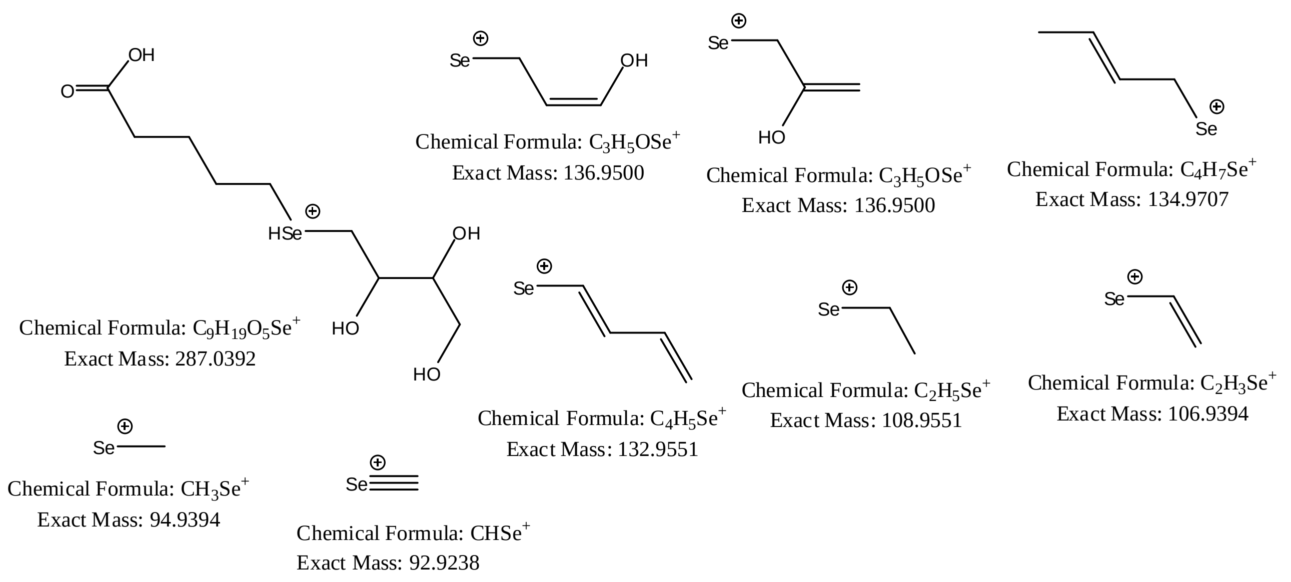

Suppl. Fig. S10 Tentative structures suggested for the $C_9H_{19}O_5Se^+$ selenospecies and its fragments obtained from the MS/MS analysis.

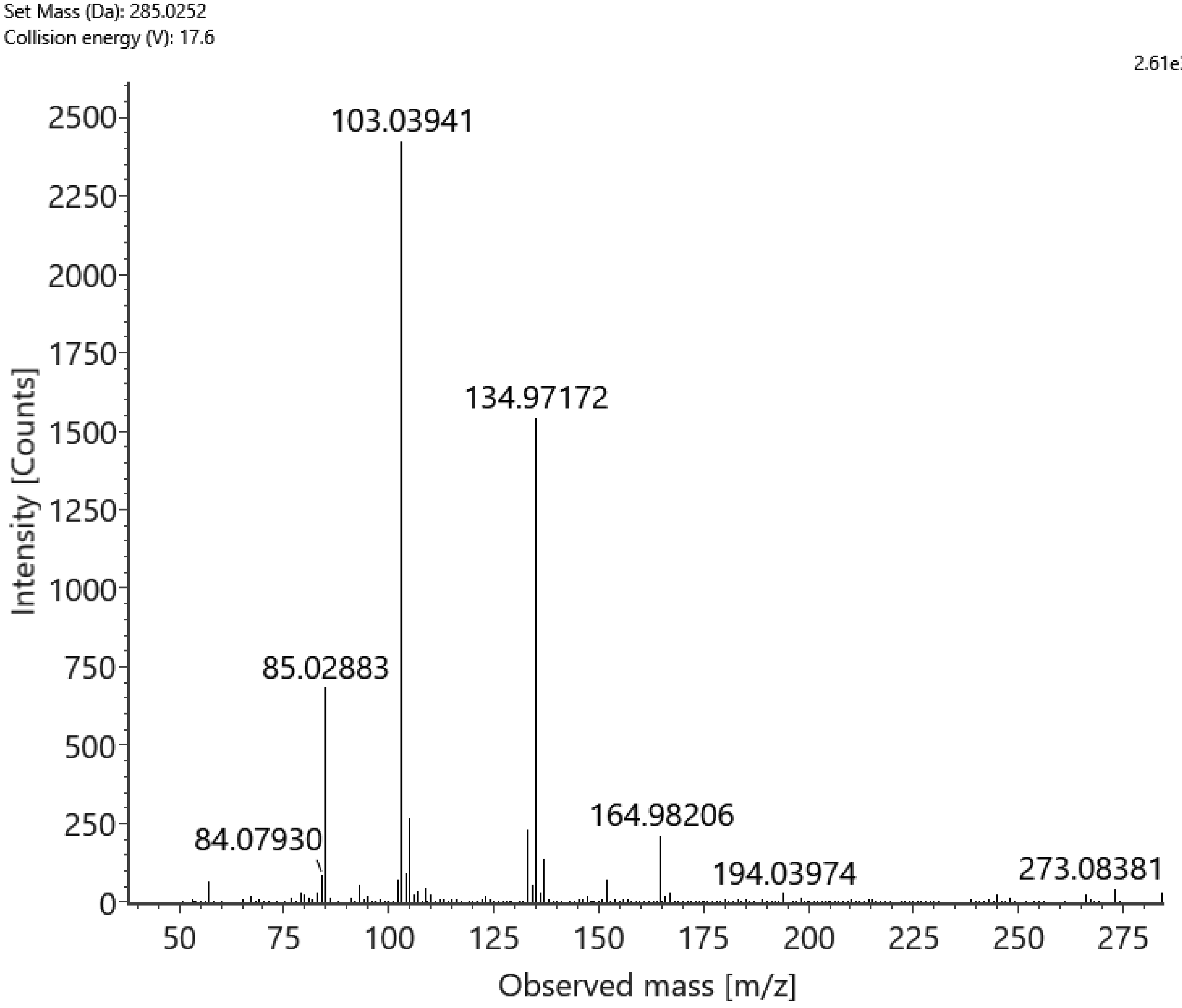

Suppl. Fig. S11 MS/MS spectrum of the $C_9H_{16}O_5Se$ (neutral state; theoretical *m/z* 285.02357 for $C_9H_{17}O_5Se^+$) selenocompound recorded in the ESI '+' mode at 17.6 eV collision energy setting.

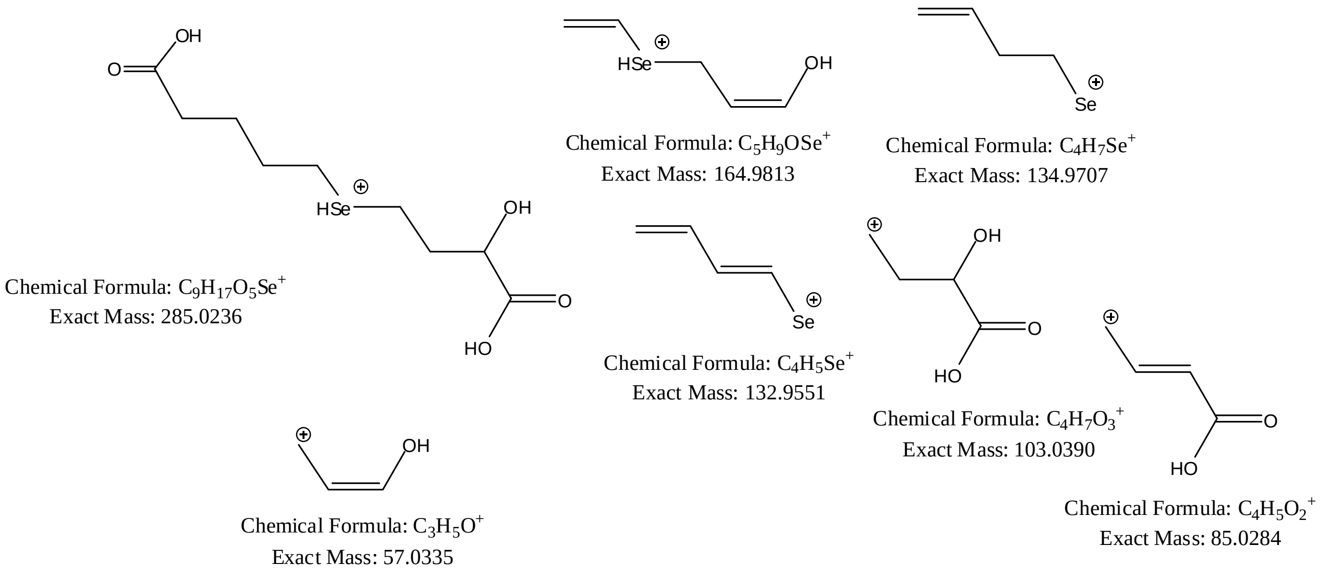

Suppl. Fig. S12 Tentative structures suggested for the $C_9H_{17}O_5Se^+$ selenospecies and its fragments obtained from the MS/MS analysis.
