# Supplementary material for: Assignment of low-molecular-weight selenometabolites in the root section of white cabbage
Source: Planta. 2025 Mar 1;261(4):71. doi: 10.1007/s00425-025-04651-y (PMC11872985; doi:10.1007/s00425-025-04651-y)
Supplement: Supplementary file 2 — Supplementary file2 (PDF 713 KB) [file 425_2025_4651_MOESM2_ESM.pdf]

ELECTRONIC SUPPLEMENTARY MATERIAL

for the paper entitled

Assignment of low molecular weight selenometabolites in the root section of white cabbage

published in

Planta

(DOI : 10.1007/s00425-025-04651-y)

authored by

Áron Soós[1], Béla Kovács[1], Tünde Takács[2], Márk Rékási[2], Péter Dobosy[3], Csaba Szőke[4], Mihály Dernovics[5]*, Péter Ragályi[2]

[1]Institute of Food Science, Faculty of Agricultural and Food Sciences and Environmental Management, University of Debrecen, Böszörményi út 138, Debrecen, 4032, Hungary

[2]Institute for Soil Sciences, HUN-REN Centre for Agricultural Research, Fehérvári út 132-144, Budapest H-1116, Hungary

[3]Institute of Aquatic Ecology, HUN-REN Centre for Ecological Research, Karolina út 29, Budapest H-1113, Hungary

[4]Department of Maize Breeding, Agricultural Institute, HUN-REN Centre for Agricultural Research, Brunszvik u. 2, Martonvásár H-2462, Hungary

[5]Department of Plant Physiology and Metabolomics, Agricultural Institute, HUN-REN Centre for Agricultural Research, Brunszvik u. 2, Martonvásár H-2462, Hungary

*corresponding author's e-mail address: dernovics.mihaly@atk.hun-ren.hu

Supplementary Table S1 Selected properties of the soils used in the experiment.

Properties	Sand	Silty sand	Silt
**pH-H_2O**	7.96	6.83	7.34
**OM (%)**	0.91	1.24	2.12
**CEC (Na meq 100 g^{-1})**	9	17	37
**$CaCO_3$ (%)**	1.45	0.08	0.20
**Total N (%)**	0.064	0.092	0.135
**AL-K (mg kg^{-1})**	61.2	144	145
**Total K (mg kg^{-1})**	3164	7250	7639
**AL-P (mg kg^{-1})**	57.2	104	35.4
**Total P (mg kg^{-1})**	449	446	412
**Water-soluble Se (mg kg^{-1})**	< LOD	< LOD	< LOD
**LE-Se (mg kg^{-1})**	0.009	0.016	0.010
**Total Se (mg kg^{-1})**	0.076	0.094	0.132
**Clay (<0.002 mm, %)**	14	23	34
**Silt (0.002-0.02 mm, %)**	18	30	50
**Sand (0.02-2 mm, %)**	69	46	16

Supplementary Table S2 UPLC-PDA-Unispray-QTOF-MS instrumental setup parameters with gradient #1.

**Acquity I-Class UPLC**		**Vion PDA IMS Unispray (+/-) -QTOF-MS**	
Eluent "A"	water with 0.1 % v/v formic acid	Source temperature	120°C
		Desolvation temperature	550°C
Eluent "B"	acetonitrile with 0.1 % v/v formic acid	Desolvation gas	1000 L/h
Flow rate	0.4 ml/min	Cone gas	100 L/h
Column temperature	25°C	IMS<br>MS scan<br>MS scan time<br>Lock mass	OFF<br>50 – 1000 m/z<br>0.2 s<br>ON
Gradient	0 – 1.0 min 10% "B"<br>1.0 – 4.0 min ↑80% "B"<br>4.0 – 4.5 min 80% "B"<br>4.5 – 5.0 min ↓10% "B"<br>5.0 – 7.0 min 10% "B"	MS^E Low collision energy	6.0 eV
Injection volume	3.0 µl	MS^E High collision energy ramp	20 – 30 eV
Sample temperature	8°C	Capillary voltage	0.3 kV
		Cone voltage	40 V
		PDA sampling rate	20 points/sec
		Wavelength	220-500 nm
		Resolution	1.2 nm

Supplementary Table S3 UPLC-PDA-Unispray-QTOF-MS instrumental setup parameters with gradient #2.

**Acquity I-Class UPLC**		**Vion PDA IMS Unispray (+/-) -QTOF-MS**	
Eluent “A”	water with 0.1 % v/v formic acid	Source temperature	120°C
		Desolvation temperature	550°C
Eluent “B”	acetonitrile with 0.1 % v/v formic acid	Desolvation gas	1000 L/h
Flow rate	0.4 ml/min	Cone gas	100 L/h
Column temperature	25°C	IMS<br>MS scan<br>MS scan time<br>Lock mass	OFF<br>100 – 2000 m/z<br>0.4 s<br>ON
Gradient	0 – 0.5 min 6% “B”<br>0.5 – 10.0 min ↑80% “B”<br>10.0 – 10.5 min 80% “B”<br>10.5 – 11.0 min ↓6% “B”<br>11.0 – 13.0 min 6% “B”	MS^E Low collision energy	6.0 eV
Injection volume	3.0 µl	MS^E High collision energy ramp	20 – 30 eV
Sample temperature	8°C	Capillary voltage	0.5 kV
		Cone voltage	40 V
		PDA sampling rate	20 points/sec
		Wavelength	220-500 nm
		Resolution	1.2 nm
